# Sigmoid resection with primary anastomosis versus the Hartmann’s procedure for perforated diverticulitis with purulent or fecal peritonitis: a systematic review and meta-analysis

**DOI:** 10.1007/s00384-020-03617-8

**Published:** 2020-06-05

**Authors:** Daniël PV Lambrichts, Pim P Edomskis, Ruben D van der Bogt, Gert-Jan Kleinrensink, Willem A Bemelman, Johan F Lange

**Affiliations:** 1 Department of Surgery, Erasmus University Medical Center, Rotterdam, 3015 GD The Netherlands; 2 Department of Surgery, Amsterdam University Medical Center, AMC, Amsterdam, The Netherlands; 3 Department of Gastroenterology and Hepatology, Erasmus University Medical Center, Rotterdam, The Netherlands; 4 Department of Neuroscience, Erasmus University Medical Center, Rotterdam, The Netherlands; 5 Department of Surgery, IJsselland Hospital, Capelle aan den IJssel, The Netherlands

**Keywords:** Perforated diverticulitis, Peritonitis, Hartmann’s procedure, Primary anastomosis

## Abstract

**Purpose:**

The optimal surgical approach for perforated diverticulitis with purulent or fecal peritonitis (Hinchey grade III or IV) remains debated. In recent years, accumulating evidence comparing sigmoid resection with primary anastomosis (PA) with the Hartmann’s procedure (HP) was presented. Therefore, the aim was to provide an updated and extensive synthesis of the available evidence.

**Methods:**

A systematic search in Embase, MEDLINE, Cochrane, and Web of Science databases was performed. Studies comparing PA to HP for adult patients with Hinchey III or IV diverticulitis were included. Data on mortality, morbidity, stoma reversal, and patient-reported and cost-related outcomes were extracted. Random effects models were used to pool data and estimate odds ratios (ORs).

**Results:**

From a total of 1560 articles, four randomized controlled trials and ten observational studies were identified, reporting on 1066 Hinchey III/IV patients. Based on trial outcomes, PA was found to be favorable over HP in terms of stoma reversal rates (OR 2.62, 95% CI 1.29, 5.31) and reversal-related morbidity (OR 0.33, 95% CI 0.16, 0.69). No differences in mortality (OR 0.83, 95% CI 0.32, 2.19), morbidity (OR 0.99, 95% CI 0.65, 1.51), and reintervention rates (OR 0.90, 95% CI 0.39, 2.11) after the index procedure were demonstrated. Data on patient-reported and cost-related outcomes were scarce, as well as outcomes in PA patients with or without ileostomy construction and Hinchey IV patients.

**Conclusion:**

Although between-study heterogeneity needs to be taken into account, the present results indicate that primary anastomosis seems to be the preferred option over Hartmann’s procedure in selected patients with Hinchey III or IV diverticulitis.

**Electronic supplementary material:**

The online version of this article (10.1007/s00384-020-03617-8) contains supplementary material, which is available to authorized users.

## Introduction

Up to 35% of patients with acute diverticulitis present with complicated disease, such as perforation with purulent or fecal peritonitis (Hinchey III or IV) [1–4]. Treatment of perforated diverticulitis with peritonitis generally requires emergency surgical treatment [5]. However, the optimal surgical treatment strategy remains a topic of debate.

Although the Hartmann’s procedure (HP) has been the favored approach for most surgeons, outcomes of sigmoidectomy with primary anastomosis (PA) have been reported to be comparable to those of HP [6, 7]. Previous studies have found PA to be associated with higher stoma reversal rates and another important potential benefit of PA is the option to avoid a defunctioning ileostomy in selected cases [8–11]. Moreover, restoration of intestinal continuity after HP is reported to be associated with higher morbidity and mortality rates [12, 13]. Hence, PA has the potential benefit to decrease patient burden, lower associated healthcare costs, and improve patient-reported outcomes [14].

Particularly in the light of increased incidence and admission rates of perforated diverticulitis, a critical appraisal of treatment strategies and their outcomes is an important step towards consensus on its optimal surgical approach [15]. Therefore, the aim of this systematic review and meta-analysis was to assess outcomes of HP and PA (with or without ileostomy) for perforated diverticulitis with purulent or fecal peritonitis.

## Methods

The study was conducted following the MOOSE and PRISMA guidelines [16, 17] and was registered in PROSPERO (CRD42019135333). Approval of the institutional review board and written consent were not required.

### Study design

Case reports, review articles, meta-analyses, letters, abstracts, or comments were excluded. Randomized controlled trials (RCTs) and prospective or retrospective cohort studies were included if they met the following criteria: reporting on (1) patients ≥ 18 years of age with acute left-sided perforated diverticulitis with peritonitis (Hinchey III or IV) and (2) a comparison of HP and PA (with or without defunctioning ileostomy). Exclusion criteria were (1) studies reporting on Hinchey I or II diverticulitis, chronic diverticular complications (e.g. fistulae or obstruction), non-diverticular colorectal disease, or elective surgery, in which outcomes could not be assessed separately from Hinchey III and IV diverticulitis; (2) non-comparative studies; and (3) non-English studies.

### Systematic literature search

A biomedical information specialist performed a systematic search in collaboration with one of the reviewers (DL). The Embase, MEDLINE, Cochrane, and Web of Science databases were searched on June 17, 2019. Publication date was not limited and the initial search was not restricted by language. Search syntaxes and results per database are given in the Appendix. An additional search through reference lists was performed. Two researchers (DL and PE) independently reviewed the identified articles by title and abstract and, subsequently, by full text using EndNote X9®. Differences in article selection were discussed and articles were included or excluded after consensus was reached between reviewers.

### Data collection

Two researchers (DL and PE) extracted data, which were checked by a third independent researcher (RB). Discrepancies were discussed until consensus was reached. In the case of uncertainties with regard to reported outcomes, corresponding authors were contacted when possible. The following study details were collected: author, year, country/countries, design, and length of follow-up, and—if applicable—sample size, inclusion period, number of screened and included patients, eligibility criteria, cross-overs, moment of randomization, primary endpoint, and trial accrual. Extracted baseline patient and operative characteristics were sex, age, body mass index (BMI), American Society of Anesthesiologists (ASA) score, preoperative disease severity, Hinchey grade, previous diverticulitis and abdominal surgery, surgical expertise, time and duration of surgery, blood loss, approach (open/laparoscopic), anastomotic configuration and construction, drain placement, and intraoperative lavage. Moreover, the following outcomes were collected: mortality, morbidity, hospital stay, intensive care unit (ICU) stay, (ongoing) sepsis, anastomotic leakage, intra-abdominal abscess occurrence and drainage, malignancies, surgical site infections (SSI), organ dysfunction, fascial dehiscence, stoma reversal rates, and hernia rates. Additionally, data on patient-reported outcomes and associated costs were extracted.

### Risk of bias and quality assessment

Study quality was assessed independently by two researchers (DL and PE) using the level of evidence [18], Newcastle-Ottawa Scale (NOS), and methodological index for non-randomized studies (MINORS) criteria [19, 20]. For RCTs, the Cochrane Collaboration’s risk-of-bias tool was used [21]. Discrepancies in quality assessment outcomes were resolved by discussion.

### Data synthesis and statistical analysis

To calculate pooled odds ratios (ORs) with 95% CI, the Mantel-Haenszel random effects model was used, which takes between-study and within-study variance into account. For continuous variables, inverse variance-weighted random effects models were used to calculate mean differences (MD) with 95% CI. Statistical heterogeneity was evaluated by calculating Q statistics and *I*^2^. In addition, risk differences (RDs), risk ratios (RRs), and numbers needed to treat (NNTs) were calculated for outcomes that were significantly different between treatment groups. Analyses were performed using RevMan 5.3 (Cochrane Centre, Copenhagen, Denmark).

## Results

### Systematic literature search

**Fig. 1:**
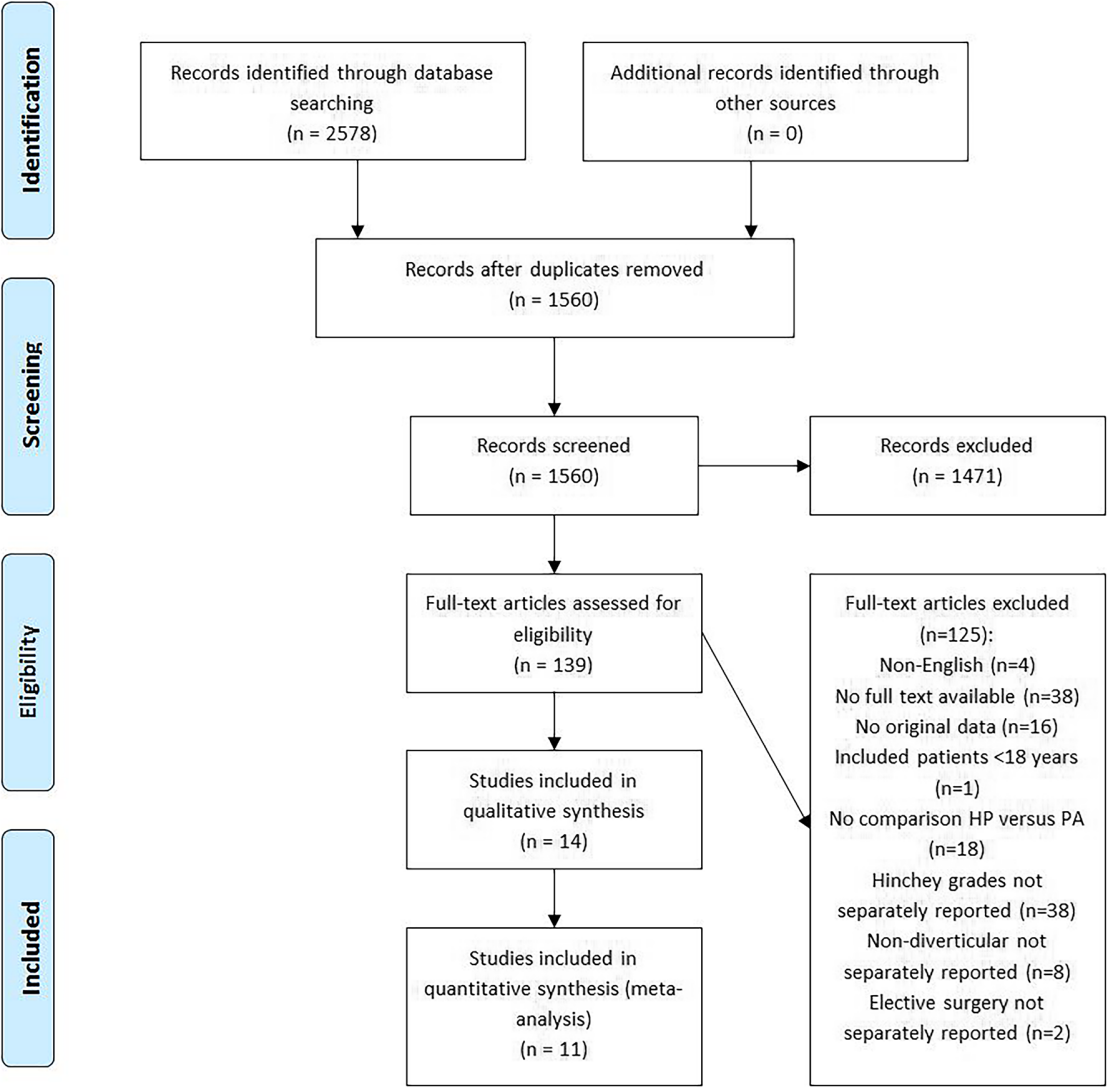
PRISMA flow diagram

Details of the study selection are provided in a PRISMA flow diagram (Fig. 1). After duplicate removal, 1560 of 2578 articles were further assessed. Eventually, 14 articles were included after title and abstract screening and full-text reading.

### Study, patient, and operative characteristics

Study characteristics are given in Table 1. Overall, four RCTs were included [22–25], as well as three prospective [10, 11, 26] and seven retrospective observational studies [27–33]. Overall, data on a total of 1274 patients were available most of whom had Hinchey grade III/IV diverticulitis (1066/1274, 83.7%). Data were available on 731 and 536 patients who underwent or were allocated to HP or PA, respectively. Risk of bias assessment of the included RCTs is shown in Supplemental Fig. 1. For the non-randomized studies, the NOS and MINORS scores ranged between 6–9 and 13–18, respectively. An overview of patient baseline characteristics is given in Table 2. Moreover, Supplemental Tables 1 and 2 provide details on the reported operative characteristics of index and reversal procedures. In Supplemental Table 3, summarized results of a quantitative analysis of baseline characteristics in the included observational studies are presented. As compared to HP, PA patients were more likely to undergo surgery for Hinchey III diverticulitis (OR 2.45, 95% CI 1.30, 4.63, *p* = 0.006) and to have a lower mean age (MD − 4.84, 95% CI − 9.41, − 0.27, *p* = 0.04) and MPI score (MD − 3.58, 95% CI − 5.70, − 1.47, *p* = 0.0009).

**Table 1:** Study characteristics

Author	Year	Country	Participating centers (*n*)	Study design	LoE	NOS	MINORS	Study period	Screened patients (*n*)	Included patients (*n*)	HP (*n*)	PA (*n*)	Hinchey grades (I/II/III/IV)	Follow-up duration	Lost to follow-up (*n*)	Reporting on stoma reversal
Randomized controlled trials																
Binda	2012	Italy, Turkey, Norway, France, Slovenia, Spain, Poland, Israel	14	RCT	1b	n.a.	n.a.	2001–2010	90	90	56	34	0/0/75/15	Until 30 days after reversal	4 (after reversal)	Yes
Bridoux	2017	France	7	RCT	1b	n.a.	n.a.	June 2008–May 2012	n.r.	102	52	50	0/0/82/20	18 months	0	Yes
Lambrichts	2019	Belgium, Italy, the Netherlands	42	RCT	1b	n.a.	n.a.	July 2010–February 2013; June 2013–June 2016	n.r.	133 (130 mITT)	66	64	0/0/92/38	12 months	1	Yes
Oberkofler	2012	Switzerland	4	RCT	1b	n.a.	n.a.	2006–2009	83 eligible (+52 not assessed for eligibility)	62	30	32	0/0/47/15	Median 47 months (38–55)	0	Yes
Observational studies																
Gooszen	2001	the Netherlands	1	Retro	2b	6	15	1979–1993	277	60	28	32	12/8/32/8	n.r.	n.r.	Yes
Mueller	2011	Germany	1	Retro	2b	6	13	1996–2006	787	73	26	47	26/33/11/3	n.r.	n.r.	No
Regenet	2003	France	1	Pro	2b	7	18	January 1994–November 2001	60	60	33	27	0/0/48/12	12 months	n.r.	Yes (colostomy)
Richter	2006	Germany	1	Retro	2b	6	14	August 2001–August 2003	215	41	5	36	III/IV: numbers not reported	n.r.	n.r.	Yes
Schilling	2001	Switzerland	1	Retro	2b	6	15	January 1994–January 1998	n.r.	55	42	13	0/0/43/12	n.r.	n.r.	Yes
Thaler	2000	Austria	1	Pro	2b	6	13	1988–1998	82	82	62	20	III/IV: numbers not reported	n.r.	n.r.	No
Trenti	2011	Spain	1	Pro	2b	9	18	January 1995–December 2008	234	87	60	27	0/0/72/15	n.r.	n.r.	Yes
Vennix	2016	the Netherlands	28	Retro	2b	9	17	July 2010–July 2014	474	Total, 307; PM, 117	Total, 240; PM, 77	Total, 67; PM, 40	PM, 0/0/96/21	LS (PM), 8 (5–12) months OS (PM), 16 (7–28 months)	n.r.	Yes
Vermeulen	2007	the Netherlands	4	Retro	2b	8	17	January 1995–January 2005	200	200	139	61	35/44/83/38	n.r.	n.r.	No
Wright	2016	the United States	2	Retro	2b	8	17	July 2006–June 2013	n.r.	115	55	53	18/32/51/14	n.r.	n.r.	Yes

**Figure:**
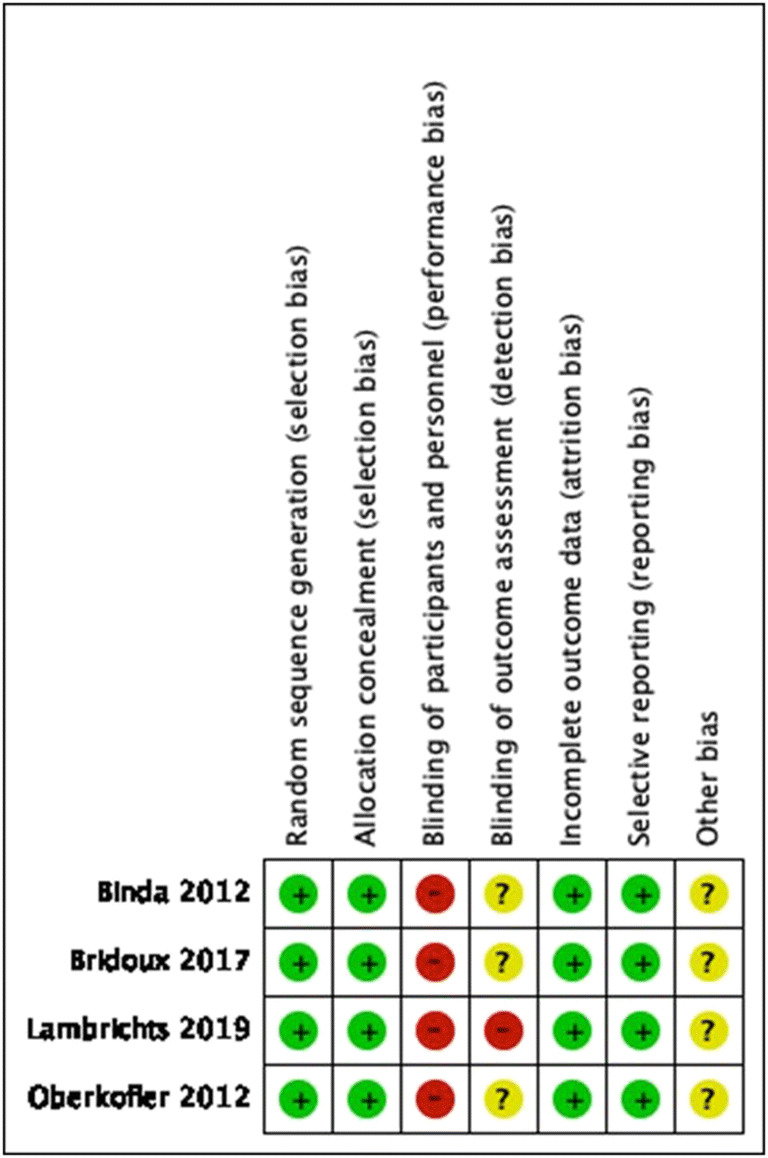
Continuous data are median (interquartile range), mean (standard deviation), or mean (range). *HP*, Hartmann’s procedure; *LoE*, level of evidence; *LS*, laparoscopic sigmoidectomy; *MINORS*, methodological index for non-randomized studies; *mITT*, modified intention-to-treat analysis; *n.a*., not applicable; *NOS*, Newcastle-Ottawa Scale; *n.r.*, not reported; *OS*, open sigmoidectomy; *PA*, primary anastomosis; *PM*, propensity-matched cohort; *Pro*, prospective study; *Retro*, retrospective study; *RCT*, randomized controlled trial

**Table 2:** Patient characteristics

Study	Group	Patients (*n*)	Sex (M/F (%))	Age (years)	BMI (kg/m^2^)	ASA (*n* (%))	Hinchey III/IV (*n* (%))	MPI	CRP (mg/l)	WBC (10^3^/μl)	Previous diverticulitis (*n* (%))	Previous abdominal surgery (*n* (%))
Randomized controlled trials												
Binda	HP	56	27/29 (48.2/51.8)	65.7 (1.8)	n.r.	n.r.	45/11 (80.4/19.6)	12.7 (0.6)	n.r.	n.r.	n.r.	15 (26.8)
	PA	34	22/12 (64.7/35.3)	63.5 (2.2)	n.r.	n.r.	30/4 (88.2/11.8)	11.4 (0.6)	n.r.	n.r.	n.r.	4 (11.8)
Bridoux	HP	52	23/29 (44.2/55.8)	61.5 (29–92)	26.8 (19.3–44.6)	> ASA I, 43 (82.7)	40/12 (76.5/23.5)	27 (20–43)	n.r.	n.r.	n.r.	n.r.
	PA	50	28/22 (56/44)	61 (25–93)	26.1 (20–43)	> ASA I, 45 (90)	42/8 (84/16)	26 (16–39)	n.r.	n.r.	n.r.	n.r.
Lambrichts	HP	66	41/25 (62/38)	61.7 (11.4)	28 (4.7)	I–II, 37 (63) III–IV, 22 (37)	46/20 (70/30)	23 (17–27)	≤ 10, 4 (6%); 11–100, 15 (23%); 101–200, 13 (20%); 201–300, 19 (29%); 301–400, 8 (12%); 401–500, 4 (6%); > 500 2 (3%); missing, 1 (2%)	14.6 (10.2–20.6)	12 (18%)	Previous laparotomy, 3 (5%)
	PA	64	41/23 (64/36)	62.4 (13.1)	26.3 (4.8)	I–II, 45 (76) III–IV, 14 (24)	46/18 (72/28)	21 (17–26)	≤ 10, 6 (9%); 11–100, 13 (20%); 101–200, 15 (23%); 201–300, 10 (16%); 301–400, 9 (14%); 401–500, 6 (9%); > 500 3 (5%); missing, 2 (3%)	14.2 (9.0–16.9)	12 (19%)	Previous laparotomy, 1 (2%)
Oberkofler	HP	30	9/21 (39/70)	74 (61–81)	24 (22–29)	I–III/V, 22/8 (73/27)	23/7 (77/23)	22 (16–28)	236 (136–307)	13 (9–17)	n.r.	n.r.
	PA	32	12/20 (38/62)	72 (60–83)	24 (23–28)	I–III/V, 24/8 (75/25)	24/8 (75/25)	24 (19–28)	194 (67–291)	13 (9–16)	n.r.	n.r.
Observational studies												
Gooszen	HP	28	n.r.	68 (16)	n.r.	n.r.	13/6 (46.4/21.4)	n.r.	n.r.	n.r.	n.r.	n.r.
	PA	32	n.r.	63 (17)	n.r.	n.r.	19/2 (59.4/6.3)	n.r.	n.r.	n.r.	n.r.	n.r.
Mueller	HP	26	10/16 (38/62)	67 (13)	n.r.	I (8%), II (16%), III (42%), IV (34%)	9/3 (35/11)	n.r.	n.r.	n.r.	n.r.	n.r.
	PA	47	26/21 (53/47)	63 (12)	n.r.	I (36%), II (33%), III (25%), IV (6%)	2/0 (4/0)	n.r.	n.r.	n.r.	n.r.	n.r.
Regenet	HP	33	18/15 (54.5/45.5)	67.3 (12.9)	n.r.	I (3%), II (51.5%), III (39.4%), IV (6.1%)	24/9 (72.7/27.3)	24.5 (6.7)	182 (130)	13.5 (7.3)	n.r.	n.r.
	PA	27	10/17 (37/63)	64.8 (16.5)	n.r.	I (3.7%), II (48.2%), III (44.4%), IV (3.7%)	24/3 (88.9/11.1)	21.2 (5.8)	133 (131)	15.3 (6.5)	n.r.	n.r.
Richter	Overall	41	19/22 (46.3/53.7)	60 (2)	n.r.	2.7 (0.2)	n.r.	20.5 (1.5)	127 (22)	n.r.	n.r.	n.r.
	HP	5	n.r.	n.r.	n.r.	n.r.	n.r.	n.r.	n.r.	n.r.	n.r.	n.r.
	PA	36	n.r.	n.r.	n.r.	n.r.	n.r.	n.r.	n.r.	n.r.	n.r.	n.r.
Schilling	HP	42	20/22 (47.6/52.4)	68 (10)	n.r.	2.5 (2–3)	33/9 (78.6/21.4)	23 (8)	159 (68)	n.r.	n.r.	n.r.
	PA	13	6/7 (46.2/53.8)	65 (12)	n.r.	2.5 (2–3)	10/3 (76.9/23.1)	21 (7)	139 (77)	n.r.	n.r.	n.r.
Thaler	HP	62	25/37 (40.3/59.7)	72 (15)	n.r.	IV/V, 44 (71)	n.r.	23 (8)	n.r.	n.r.	n.r.	n.r.
	PA	20	6/14 (30/70)	70 (13)	n.r.	IV/V, 7 (35)	n.r.	18 (7)	n.r.	n.r.	n.r.	n.r.
Trenti	HP	60	34/26 (56.7/43.3)	69.7 (12.7)	n.r.	I (6.7%), II (13.3%), III (33.3%), IV (46.7%)	46/14 (76.7/23.3)	n.r.	n.r.	n.r.	n.r.	n.r.
	PA	27	19/8 (70.4/29.6)	58.1 (16.3)	n.r.	I (29.6%), II (51.9%), III (18.5%), IV (0%)	26/1 (96.3/3.7)	n.r.	n.r.	n.r.	n.r.	n.r.
Vennix	Overall (open)	263	138/125 (52.5/47.5)	62.6 (13.9)	27.0 (5.8)	I (16.5%), II (45.6%), III (32.3%), IV (5.7%)	173/90 (65.3/34.7)	21.1 (5.6)	221 (142)	14.7 (11.4)	50 (19.1)	26 (9.9)
	Overall (laparosocopic)	44	30/14 (68.2/31.8)	56.2 (13.5)	25.6 (3.9)	I (24.2%), II (39.4%), III (33.3%), IV (3%)	34/10 (76.7/23.3)	19.1 (5.4)	156 (114)	15.2 (8.7)	7 (16.7)	1 (2.3)
	HP	240	n.r.	n.r.	n.r.	n.r.	n.r.	n.r.	n.r.	n.r.	n.r.	n.r.
	PA	67	n.r.	n.r.	n.r.	n.r.	n.r.	n.r.	n.r.	n.r.	n.r.	n.r.
Vermeulen 2007	HP	139	64/75 (46/54)	69 (13)	n.r.	I (18%), II (22%), III (33%), IV (27%)	62/33 (45/24)	21 (8)	n.r.	n.r.	n.r.	n.r.
	PA	61	25/36 (41/59)	62 (15)	n.r.	I (28%), II (31%), III (30%), IV (11%)	21/5 (34/8)	17 (6)	n.r.	n.r.	n.r.	n.r.
Wright	Overall (colorectal)	62	25/37 (40.3/59.7)	62.7 (13.3)	28.4 (16–51.3)	I (3.2%), II (41.9%), III (45.2%), IV (9.7%)	20/7 (32.3/11.3)	n.r.	n.r.	n.r.	36 (58.1)	n.r.
	Overall (general)	53	27/26 (50.9/49.1)	63.4 (14.4)	26.8 (18.9–54.1)	I (3.8%), II (47.2%), III (32.1%), IV (17.0%)	31/7 (58.5/13.2)	n.r.	n.r.	n.r.	13 (24.5)	n.r.
	HP	55	n.r.	n.r.	n.r.	n.r.	n.r.	n.r.	n.r.	n.r.	n.r.	n.r.
	PA	53	n.r.	n.r.	n.r.	n.r.	n.r.	n.r.	n.r.	n.r.	n.r.	n.r.

Continuous data are median (interquartile range), mean (standard deviation), or mean (range). *ASA*, American Society of Anesthesiologists; *BMI*, body mass index; *CRP*, C-reactive protein; *HP*, Hartmann’s procedure; *MPI*, Mannheim Peritonitis Index; *n.r.*, not reported; *PA*, primary anastomosis; *WBC*, white blood cell count

### Randomized controlled trials

Details of the four included RCTs are provided in Supplemental Table 4. Overall, 204 HP and 180 PA patients were analyzed. Reported inclusion and exclusion criteria varied between trials, mainly in terms of exclusion criteria. The only trial to report reasons for not screening patients for eligibility and non-inclusion of screened patients was that by Oberkofler and colleagues [23]. Overall, 53 patients were not assessed for participation due to disagreement of the surgeon (40% HP, 30% PA with diverting ileostomy, 22% PA without diverting ileostomy, 8% others). Moreover, the authors reported that 21 patients were not included, because they declined to participate (*n* = 7) or did not meet inclusion criteria (*n* = 14). The Ladies trial [25] was the only study to be able to assess differences between the included patients and a cohort of 235 non-included but eligible patients, showing that in the latter group a GI surgeon was less often present (68.7% vs. 88.5%, *p* < 0.001) and the median interval to surgery was longer (13.5 h (6–43.8) vs. 8.8 (5.3–29.3), *p* = 0.02). However, no difference in in-hospital mortality was found for non-included (20/235, 8.5%) and included patients (6/130 (4.6%), *p* = 0.21). Three of the four trials randomized preoperatively, whereas in the Ladies trial patients were randomized intraoperatively. All trials were terminated early due to recruitment difficulties. Oberkofler et al. [23] reported significant differences in relevant secondary endpoints to be an additional argument for early discontinuation, although they did not specify which endpoints. In total, 31.5% (384/1218) of the overall calculated sample sizes was reached.

### Index procedure: mortality

**Fig. 2:**
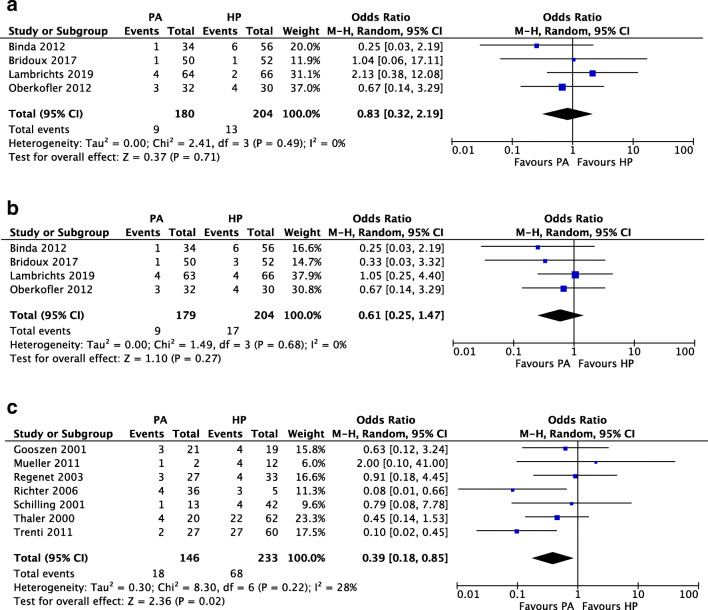
Quantitative analyses of **a** short-term mortality rates in randomized controlled trials, **b** long-term mortality rates in randomized controlled trials, and **c** overall mortality rates during follow-up in observational studies

An overview of outcomes after the index procedure is given in Tables 3 and 4. Eleven of the included studies reported on mortality rates during follow-up for Hinchey III/IV patients. As shown in Fig. 2a, no difference was found in the occurrence of short-term mortality in a quantitative analysis of RCTs, with mortality occurring in 5% (9/180) of PA and 6.4% (13/204) of HP patients (OR 0.83 (95% CI 0.32, 2.19)). In addition, long-term mortality, defined as occurring within the trials’ full study period, showed no difference between PA and HP (9/179 (5%) vs. 17/204 (8.3%), OR 0.61, 95% CI 0.25, 1.47) as shown in Fig. 2b. A separate quantitative analysis of data from observational studies (*n* = 7) showed a significant difference in overall mortality in favor of PA (18/146 (12.3%)) as compared to HP (68/233 (29.2%)) with an OR of 0.39 (95% CI 0.18, 0.85) (Fig. 2c).

**Table 3:** Outcomes of the index operation (1/2)

Study	Group	Patients (*n*)	Mortality	Mortality period	Morbidity	Morbidity period	Clavien-Dindo	Clavien-Dindo period	Reinterventions	LOS (days)	ICU stay
Randomized controlled trials											
Binda	HP	56	6 (10.7%)	30-day	26 (46.4%)^a^	30-day	n.r.	n.a.	1 (1.8%)	n.r.	n.r.
	PA	34	1 (2.9%)	30-day	12 (35.3%)^a^	30-day	n.r.	n.a.	1 (2.9%)	n.r.	n.r.
Bridoux	HP	52	1 (3.8%)	Postoperative period	22 (42.3%)^a^	n.s.	7 (13.5%)^b^	n.s.	2 (3.8%) abscess	11 (4–88)	9.5 (3–27)
	PA	50	1 (2%)	Postoperative period	27 (54%)^a^	n.s.	7 (14%)^b^	n.s.	1 (2%) abscess, 2 (4%) anastomotic leakage	11.5 (3–53)	9.5 (1–71)
Lambrichts	HP	66	2 (3%)	In-hospital/30 days	Major, 8 (12%)^c^ Minor, 26 (39%)^c^ Overall, 29 (44%)^c^	In-hospital/30 days	12 (18%)^d^	90-day	4 (6%) surgical	9.0 (7–15)	2.0 (1–11)
	PA	64	4 (6%)	In-hospital/30 days	Major, 9 (14%)^c^ Minor, 19 (30%)^c^ Overall, 25 (39%)^c^	In-hospital/30 days	9 (14%)^d^	90-day	4 (6%) surgical	9.5 (7–13)	1.5 (1–3)
Oberkofler	HP	30	4 (13%)	In-hospital	Overall^f^, 24 (80%)	n.s.	12 (40%)^d^	n.s.	n.r.	18 (14–27)	2 (1–3)
	PA	32	3 (9%)	In-hospital	Overall^f^, 27 (84%)	n.s.	14 (44%)^d^	n.s.	n.r.	16 (13–25)	1 (1–4)
Observational studies											
Gooszen	HP	Overall, 28 Hinchey III/IV, 19	4/19 (21.1%)	In-hospital	n.r.s.	n.r.s.	n.r.s.	n.r.s.	n.r.s.	n.r.s.	n.r.s.
	PA	Overall, 32 Hinchey III/IV, 21	3/21 (11.1%)	In-hospital	n.r.s.	n.r.s.	n.r.s.	n.r.s.	n.r.s.	n.r.s.	n.r.s.
Mueller	HP	26	Hinchey III, 2 Hinchey IV, 2	In-hospital	Major^e^: Hinchey III, 3 Hinchey IV, 3	In-hospital	n.r.s.	n.r.s.	n.r.s.	n.r.s.	n.r.s.
	PA	47	Hinchey III, 1	In-hospital	Major^e^: Hinchey III, 3	In-hospital	n.r.s.	n.r.s.	n.r.s.	n.r.s.	n.r.s.
Regenet	HP	33	4 (12.1%)	In-hospital/30 days	n.r.	n.r.	n.r.	n.r.	4 (12.1%)	n.r.	9.8 (20.8)
	PA	27	3 (11.1%)	In-hospital/30 days	n.r.	n.r.	n.r.	n.r.	2 (7.4%)	n.r.	5.3 (12.4)
Richter	Overall	41	7 (17.1%)	n.s.	n.r.	n.r.	n.r.	n.r.	–	n.r.	5 (1.4)
	HP	5	3 (60%)	n.s.	n.r.	n.r.	n.r.	n.r.	0	n.r.	–
	PA	36	4 (11.1%)	n.s.	n.r.	n.r.	n.r.	n.r.	1 (2.8%)	n.r.	–
Schilling	HP	42	4 (9.5%)	n.s.	5 (11.9%) major^g^, 9 (21.4%) minor^g^	n.s.	n.r.	n.r.	n.r.	15.4 (6.6) regular ward	2.1 (5.1)
	PA	13	1 (7.7%)	n.s.	1 (7.7%) major^g^, 5 (38.5%) minor^g^	n.s.	n.r.	n.r.	n.r.	17.4 (10.9) regular ward	0.8 (1.4)
Thaler	HP	62	22 (35%)	n.s.	7 (11%) surgical, 13 (21%) overall	n.s.	n.r.	n.r.	n.r.	22 (20)	21 (37%)
	PA	20	4 (20%)	n.s.	6 (30%) surgical, 7 (35%) overall	n.s.	n.r.	n.r.	n.r.	23 (12)	5 (28%)
Trenti	HP	60	27 (45%)	n.s.	52 (86.7%)^a^	n.s.	n.r.	n.r.	12 (20%)	27.9 (22.8)	n.r.
	PA	27	2 (7.4%)	n.s.	13 (48.1)^a^	n.s.	n.r.	n.r.	3 (11.1%)	15.1 (9.4)	n.r.

Continuous data are median (interquartile range), mean (standard deviation), or mean (range). a = overall morbidity; b = Clavien-Dindo III–V; c = major morbidity defined as surgical reintervention, percutaneous abscess drainage, fascial dehiscence, urosepsis, myocardial infarction, renal failure, and respiratory insufficiency, minor morbidity defined as surgical site infection, postoperative ileus, pneumonia, delirium, urinary tract infection, abscess without drainage, thrombosis, cardiac complications, and overall morbidity defined as major and minor complications combined; d = Clavien-Dindo IIIb–V; e = major defined as anastomotic leakage, leakage of the Hartmann’s stomp, or stoma necrosis; f = Clavien-Dindo I–V; g = major complications defined as those which required change in therapy or prolonged therapy, minor not defined. *HP*, Hartmann’s procedure; *ICU*, intensive care unit; *LOS*, length of stay; *n.r.*, not reported; *n.r.s.*, not reported separately; *n.s.*, not specified; *PA*, primary anastomosis; *PM*, propensity-matched cohort

**Table 4:** Outcomes of the index operation (2/2)

Study	Group	(Ongoing) sepsis	Anastomotic leakage	Intra-abdominal abscess	Abscess drainage	SSI	Other infectious complications	Organ dysfunction	Fascial dehiscence	Incisional hernia	Parastomal hernia	Malignancy
Randomized controlled trials												
Binda	HP	n.r.	1 (1.8%) (rectal stump leak)	6 (10.7%)	6 (10.7%)	11 (19.6%) superficial, 9 (16.1%) deep	4 (7.1%) pneumonia, 3 (5.4%) UTI	6 (10.7%)	n.r.	n.r.	n.r.	1 (1.8%)
	PA	n.r.	1 (2.9%)	0	0	9 (26.5%) superficial, 6 (17.6%) deep	2 (5.9%) pneumonia	1 (2.9%)	n.r.	n.r.	n.r.	2 (5.9%)
Bridoux	HP	0	0	4 (7.7%)	2 (3.8%)	n.r.	n.r.	n.r.	n.r.	n.r.	n.r.	2 (3.8%)
	PA	1 (2%)	2 (4%)	2 (4%)	1 (2%)	n.r.	n.r.	n.r.	n.r.	n.r.	n.r.	3 (6%)
Lambrichts	HP	2 (3%)	0	7 (10.6%)	2 (3%)	8 (12%)	5 (8%) pneumonia, 2 (3%) UTI	4 (6%) respiratory failure, 3 (5%) renal failure	0	5 (8%)	10 (16%)	0
	PA	4 (6%)	0	2 (3%)	2 (3%)	7 (11%)	2 (3%) UTI	1 (2%) respiratory failure, 1 (2%) renal failure	3 (5%)	3 (5%)	0	2 (3%)
Oberkofler	HP	2 (6.7%)	0	6 (20%)*	n.r.	13 (43.3%)	3 (10%) UTI	6 (20%) respiratory; 5 (16.7%) cardiovascular; 5 (16.7%) renal failure	3 (10%)	n.r.	n.r.	n.r.
	PA	4 (12.5%)	1 (3.1%)	2 (6.3%)*	n.r.	11 (34.4%)	1 (3.1%)	7 (21.9%) respiratory; 3 (9.4%) cardiovascular; 2 (6.3%) renal failure	4 (12.5%)	n.r.	n.r.	n.r.
Observational studies												
Gooszen	HP	n.r.s.	n.r.s.	n.r.s.	n.r.s.	n.r.s.	n.r.s.	n.r.s.	n.r.s.	n.r.s.	n.r.s.	n.r.s.
	PA	n.r.s.	n.r.s.	n.r.s.	n.r.s.	n.r.s.	n.r.s.	n.r.s.	n.r.s.	n.r.s.	n.r.s.	n.r.s.
Mueller	Overall	n.r.s.	Hinchey III, 3	n.r.s.	n.r.s.	n.r.s.	n.r.s.	n.r.s.	n.r.s.	n.r.s.	n.r.s.	n.r.s.
	HP	n.r.s.	n.r.s.	n.r.s.	n.r.s.	n.r.s.	n.r.s.	n.r.s.	n.r.s.	n.r.s.	n.r.s.	n.r.s.
	PA	n.r.s.	n.r.s.	n.r.s.	n.r.s.	n.r.s.	n.r.s.	n.r.s.	n.r.s.	n.r.s.	n.r.s.	n.r.s.
Regenet	HP	2 (6.1%)	2 (6.1%)	3 (9.1%)^†^	n.r.	9 (27.3%)	n.r.	7 (21.2%) respiratory complication; 1 (3%) cardiac complication; 2 (6.1%) renal insufficiency	1 (3%)^§^	n.r.	n.r.	n.r.
	PA	2 (7.4%)	3 (11.1%)	1 (3.7%)^†^	n.r.	2 (7.4%)	n.r.	4 (14.8%) respiratory complication; 0 cardiac complication; 0 renal insufficiency	2 (7.4%)^§^	n.r.	n.r.	n.r.
Richter	HP	n.r.	0	n.r.	n.r.	n.r.	n.r.	n.r.	n.r.	n.r.	n.r.	n.r.
	PA	n.r.	1 (2.8%)	n.r.	n.r.	n.r.	n.r.	n.r.	n.r.	n.r.	n.r.	n.r.
Schilling	HP	n.r.	0	n.r.	n.r.	n.r.	n.r.	n.r.	n.r.	n.r.	n.r.	n.r.
	PA	n.r.	0	n.r.	n.r.	n.r.	n.r.	n.r.	n.r.	n.r.	n.r.	n.r.
Thaler	HP	n.r.	0	n.r.	n.r.	n.r.	n.r.	n.r.	n.r.	n.r.	n.r.	n.r.
	PA	n.r.	3 (15%) fascial of anastomotic dehiscence	n.r.	n.r.	n.r.	n.r.	n.r.	n.r.	n.r.	n.r.	n.r.
Trenti	HP	14 (23.3%)	0	8 (13.3%)	5 (8.3%)	19 (31.7%)	17 (28.3%) pulmonary infection/insufficiency	9 (11.4%) cardiac decompensation	7 (11.7%)^§^	n.r.	n.r.	n.r.
	PA	1 (3.7%)	3 (11.1%)	0	0	10 (37%)	1 (3.7%) pulmonary infection/insufficiency	1 (3.7%) cardiac decompensation	0	n.r.	n.r.	n.r.

Continuous data are median (interquartile range), mean (standard deviation), or mean (range). *Defined as intra-abdominal infection. ^†^Defined as secondary peritonitis or intra-abdominal abscess. ^§^Defined as wound dehiscence. *HP*, Hartmann’s procedure; *n.a.*, not applicable; *n.r.*, not reported; *n.r.s.*, not reported separately; *PA*, primary anastomosis; *SSI*, surgical site infection; *UTI*, urinary tract infection

### Index procedure: morbidity

**Fig. 3:**
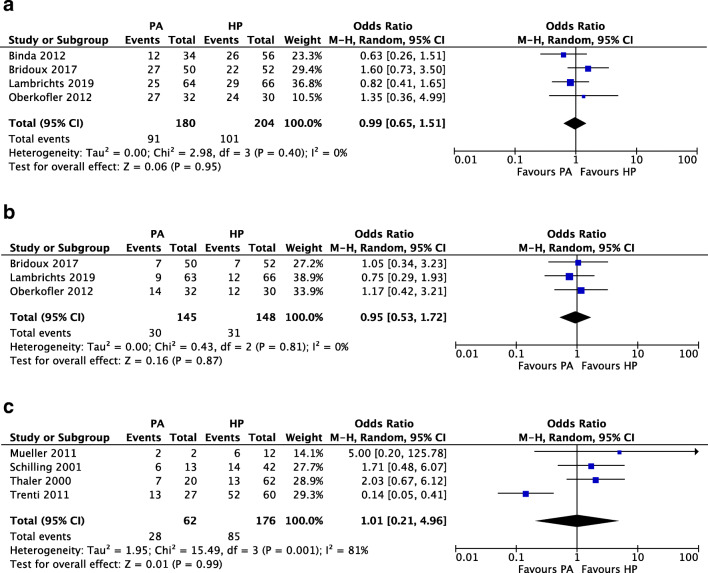
Quantitative analyses of **a** overall morbidity rates in randomized controlled trials, **b** short-term serious complications in randomized controlled trials, and **c** overall morbidity rates in observational studies

The overall morbidity rates in RCTs are provided in Fig. 3a, which shows no difference between both procedures with an OR of 0.99 (95% CI 0.65, 1.51; PA 91/180 (50.6%) vs. HP 101/204 (49.5%)). An additional analysis of short-term serious complications (Clavien-Dindo grade > IIIa) within the RCTs (Fig. 3b) also did not show a difference between PA and HP (30/145 (20.7%) vs. 31/148 (20.9%), OR 0.95, 95% CI 0.53, 1.72). Additionally, morbidity could be assessed in four observational studies (Fig. 3c), which showed an OR of 1.01 (95% CI 0.21, 4.96; PA 28/62 (45.2%) vs. HP 85/176 (48.3%)).

Reintervention rates after the index procedure, including surgical reinterventions and abscess drainage, were assessed within the RCTs (Supplemental Fig. 2a) and no differences were demonstrated between both procedures (PA 11/148 (7.4%) vs. HP 13/174 (7.5%); OR 0.90, 95% CI 0.39, 2.11). A separate analysis of reoperation rates within these trial data also showed no differences (Supplemental Fig. 2b). From observational studies (*n* = 3), reintervention rates were 6.7% (6/90) and 16.3% (16/98) for PA and HP, respectively (OR 0.52, 95% CI 0.19, 1.46; see Supplemental Fig. 2c).

Nine studies provided anastomotic leakage rates after the index procedure, which showed the occurrence of 14 leakages in 226 PA patients (6.2%) and 3 leakages in 298 HP patients (1%). In the latter group, one patient had a rectal stump leakage [22], whereas two other patients were stated to have anastomotic leakage due to the presence of fistulas in the study by Regenet et al. [10]. Forest plots of surgical site infections, postoperative (ongoing) sepsis, and fascial dehiscence did not show significant differences between both treatment groups in experimental and observational studies (Supplemental Fig. 3a, b, c, d, e, and f).

### Stoma- and reversal-related outcomes

**Fig. 4:**
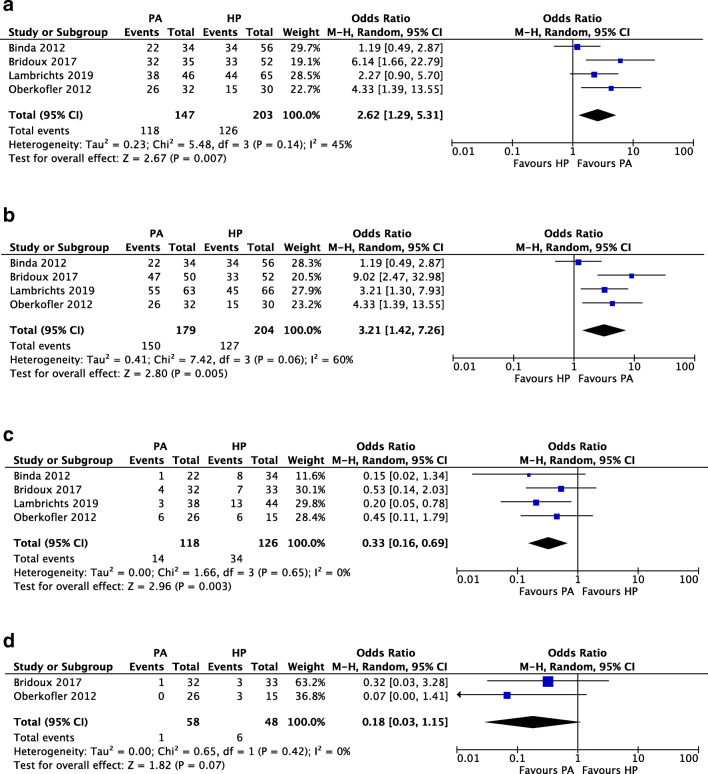
Quantitative analyses of randomized controlled trials: **a** reversal rates of constructed stomas, **b** number of stoma-free patients, **c** reversal-related morbidity, **d** reversal-related serious complications

An overview of outcomes after the reversal procedure is given in Supplemental Tables 6a and b. In Fig. 4a, reversal rates of constructed stomas were assessed within the included trials, showing a significant difference in favor of PA (118/147 (80.3%)) over HP (126/203 (62.1%); OR 2.62, 95% CI 1.29, 5.31), with an associated NNT of 5 (Supplemental Table 5). From the assessment of the number of stoma-free patients during trial follow-up, as provided in Fig. 4b, PA also showed favorable outcomes over HP (PA 150/179 (83.8%) vs. HP 127/204 (62.3%); OR 3.21, 95% CI 1.42, 7.26; NNT 5). Reversal rates of the studies that could not be included are shown in Supplemental Table 6a.

Reasons for non-reversal were mentioned in the trial of Oberkofler et al. [23], including patient’s choice and the surgeon’s risk assessment, but related percentages were not presented. Bridoux and colleagues found that reasons for not undergoing stoma reversal in the HP group were the patient’s choice (*n* = 8/17) or if a patient was deemed unfit for surgery (*n* = 9/17) [24]. The latter was also the case in 2 of 16 PA patients, whereas the other 14 patients had no stoma constructed. In the Ladies trial, reasons were the surgeon’s disapproval (HP: *n* = 3/21, PA: *n* = 1/8), patient’s preferences (HP: *n* = 2/21, PA; *n* = 1/8), or mortality before reversal (HP: 7/21, PA: *n* = 4/8), or they were unknown (HP: *n* = 8/21, PA: *n* = 2/8) [25].

One case of reversal-related mortality was reported within the included studies, which was caused by mesenteric ischemia after atrial fibrillation [24]. Postoperative morbidity related to the reversal procedure was assessed in four studies (Fig. 4c), being the four included trials, which showed a significant difference in favor of PA (PA 14/118 (11.9%) vs. HP 34/126 (27%); OR 0.33, 95% CI 0.16, 0.69; NNT 7). This difference was not found when serious complications were assessed in two of the four trials that reported these figures, as shown in Fig. 4d (PA 1/58 (1.7%) vs. HP 6/48 (12.5%); OR 0.18, 95% CI 0.03, 1.15).

### Outcomes of index and reversal procedure combined

An analysis of the short-term mortality of the index and reversal procedures combined (Supplemental Fig. 4a) did not show a significant difference, with an OR of 0.76 (PA 9/179 (5%) vs. HP 14/204 (6.9%); 95% CI 0.29, 1.96). Additionally, short-term morbidity was assessed for the combined procedures (Supplemental Fig. 4b), which showed no difference between both treatment groups (PA 88/179 (49.2%) vs. HP 120/204 (58.8%); OR 0.64, 95% CI 0.37, 1.13). The occurrence of anastomotic leakage after both the index and reversal procedure combined was assessed in the four RCTs, which did not show a difference between PA and HP (respectively, 6/179 (3.4%) vs. 6/204 (2.9%); OR 1.04, 95% CI 0.30, 3.52; see Supplemental Fig. 4c).

### Outcomes in Hinchey IV patients

Four studies specifically reported on outcomes of Hinchey IV diverticulitis. Binda et al. [22] found the type of peritonitis (purulent or fecal) to be significantly related to morbidity (28/75 (37.3%) vs. 10/15 (66.7%), *p* = 0.047) and mortality (3/75 (4%) vs. 4/15 (26.7%), *p* = 0.014) in multivariate analysis. Outcomes of Hinchey III and IV patients were assessed separately in the Ladies trial [25], showing the 12-month stoma-free survival after PA to be significantly better for both Hinchey grades (III: hazard ratio 2.35, 95% CI 1.49, 3.71; IV: hazard ratio 4.15, 95% CI 1.71, 10.1). Within the Hinchey IV group, no significant differences in short-term postoperative outcomes after the index procedure were demonstrated between HP and PA (mortality 2/20 (10%) vs. 3/18 (16.7%); overall morbidity 12/20 (60%) vs. 8/18 (44.4%), *p* = 0.52). Also, no differences in short-term post-reversal outcomes were found, with no mortality in both treatment groups and an overall morbidity of 30% in the HP group and 0% in the PA group (*p* = 0.21). Trenti and colleagues [11] performed a logistic regression analysis with Hinchey grade (IV vs. III), Peritonitis Severity Score (≤ 9), ASA (III–IV vs. I–II), and treatment (HP vs. PA), but did not find Hinchey grade to be independently associated with postoperative mortality and morbidity, wound infection, or reoperation. Moreover, in the study by Vermeulen et al. [30], multivariate analyses adjusting for treatment, age, ASA, MPI, surgeon’s experience, and Hinchey grades showed Hinchey grade IV disease to be independently associated with the outcome as compared to the reference group of Hinchey I patients (postoperative mortality: OR 3.9, 95% CI 1.0, 13.8, *p* = 0.03; reinterventions: OR 3.9, 95% CI 1.3, 12.7, *p* = 0.02).

### Outcomes of primary anastomosis with or without ileostomy

Bridoux et al. [24] stated that no related mortality was found in the subgroup of selected PA patients without an ileostomy (*n* = 15). Moreover, they reported overall morbidity and serious complication rates to be lower in PA patients without a stoma (respectively, 0% vs. 27%, *p* = 0.01, and 23% vs. 67%, *p* = 0.042). In the Ladies trial [25], PA patients with (*n* = 40) and without (*n* = 17) an ileostomy were compared. No differences in overall morbidity (4/17 (23.5%) vs. 18/40 (45%), *p* = 0.15) and mortality (0 vs. 3/40 (7.5%), *p* = 0.55) were found, but patients without an ileostomy had a significantly shorter median postoperative stay (7 (11–14) days vs. 11 (7–14), *p* = 0.01). In their overall cohort (including Hinchey I/II diverticulitis), Vermeulen and colleagues [30] found no difference in complication rates between PA patients with or without an ileostomy (respectively, 19% vs. 11%, *p* = 0.42).

### Patient-reported outcomes

Patient-reported outcomes were only identified within the Ladies trial [25]. During the 12-month follow-up period, questionnaires to measure health-related quality of life were sent out at weeks 2 and 4, and months 3, 6, and 12 after the initial procedure. The questionnaires used were the EuroQol-5D-3-level, Short Form-36v2, and Gastrointestinal Quality of Life Index. Between treatment groups, no significant differences were found in summarized scores or subscales.

### Cost-related outcomes

Two studies compared both procedures in terms of associated costs. Schilling et al. [28] compared costs (converted to US dollars) associated with operative time, intensive care and hospital stay, and other resources (e.g., antibiotics, packed red blood cells, and fresh frozen plasma), demonstrating overall expenses to be 74 to 299% higher for HP with subsequent restoration of intestinal continuity as compared to PA. Oberkofler et al. [23] reported on in-hospital costs in US dollars, but found no significant differences for the index and reversal procedure or both procedures combined. Mean (s.d.) costs associated with the combined procedures were 77.943 ± 50.352 and 75.208 ± 58.002 (*p* = 0.880) for HP and PA, respectively. A cost analysis of the DIVA arm of the Ladies trial is to be expected [25].

## Discussion

From this systematic review and meta-analysis of the available evidence on PA versus HP for perforated diverticulitis with purulent or fecal peritonitis, several arguments can be identified to support the choice of PA over HP. Firstly, no difference in mortality and morbidity after the index procedure was found between both procedures. Secondly, PA patients were more likely to have their stoma reversed and to be stoma free during follow-up, as compared to HP patients. In addition, the occurrence of reversal-related morbidity was less likely in the PA group.

Although in recent years other meta-analyses on this topic have been published, the present study included the recently published Ladies trial [25], which allowed for the analysis of a larger cohort of patients from randomized studies. Moreover, as compared to these previous review articles, a more extensive scope of outcomes (e.g., patient-reported and cost-related) and results within subgroups of interest (e.g., PA patients with or without ileostomy and Hinchey IV patients) were assessed.

The present results are generally in line with those from previous meta-analyses. With regard to overall mortality from randomized and observational studies, Gachabayov et al. [34] and Shaban et al. [35] found PA to be favorable over HP, which was also the case in the present quantitative analysis of mortality within the included observational studies. However, in the subgroup analysis of randomized studies in the present study, no difference between treatment groups was demonstrated, which is comparable to the outcomes found by Acuna et al. [36]. Furthermore, Acuna et al. [36] and Gachabayov et al. [34] both demonstrated PA patients to be more likely to undergo stoma reversal and be stoma free during follow-up, which is similar to the present results. Recently, two population-based analyses of patients who underwent emergency surgery for acute diverticulitis in the USA were published by Lee et al. [37] and Gawlick et al. [38]. Although these studies were not included in this meta-analysis due to the lack of specification on Hinchey grades of included patients, their outcomes provide context to the present findings. The authors show favorable outcomes with regard to mortality after PA as compared to HP and did not find differences in complication rates. Interestingly, a third US population-based study by Cauley et al. [39] concluded less favorable on the role of PA. Most importantly, all three studies corrected for potential confounders by means of multivariable regression analyses incorporating factors such as age, BMI, ASA grade, and severity of sepsis. The importance of these potential confounders must be emphasized, as treatment outcomes might be subject to confounding by indication and, thereby, influence the generalizability and interpretation of the present results. Notably, from the quantitative synthesis of baseline characteristics within the included observational studies, this present study indeed found that PA patients were more likely to be younger and have less severe disease in terms of Hinchey grade and MPI scores.

In this review, results were only used for quantitative analyses if they could be assessed specifically for Hinchey III and IV diverticulitis. This strict inclusion and analysis approach was chosen in order to strengthen our conclusion, by avoiding the chance of overestimating true treatment effects through inclusion of patients with less severe disease entities (e.g., Hinchey II diverticulitis). In addition, another strength of this study is the before-mentioned broad scope of outcomes, including cost-related and patient-reported outcomes. Diverticulitis is a costly disease and the incidence of perforated diverticulitis is increasing; therefore, insights into the treatment costs are of interest [15, 40, 41]. With benefits such as higher reversal rates and less reversal-related morbidity, PA has the potential to save both direct and indirect medical costs. However, despite its relevance, only two studies reported on the directly associated costs and, therefore, no robust conclusions could yet be drawn [23, 28], especially, since cost-effectiveness and cost-utility analyses were not described. The presence of a stoma is known to negatively affect factors such as physical function and body image, and, consequently, quality of life [14]. In this regard, the stoma-related benefits of PA might be able to improve the overall quality of life. Nevertheless, patient-related outcomes could only be identified in the Ladies trial, which showed no differences in outcomes of general and gastro-intestinal questionnaires [25]. Novel and stronger evidence could be of importance, as the potential beneficial cost-related and patient-reported outcomes could likely be valuable additional arguments to opt for PA. Moreover, it could help with its wider implementation into clinical practice, particularly, as it is suggested that HP still remains the most widely used procedure in past years [37].

A lack of evidence was identified with regard to the question whether or not it is safe to omit the construction of a defunctioning ileostomy in PA patients, and, if so, under what circumstances. Results for PA patients without an ileostomy seemed comparable to those of patients with an ileostomy, albeit that groups were small and at risk for selection bias [25]. Similarly, outcomes specifically reported for Hinchey IV patients were scarce and consisted of relatively small groups. It was demonstrated that PA had a significantly better 12-month stoma-free survival as compared to HP within Hinchey IV patients [25]. Additionally, some authors found Hinchey IV to be independently associated with an increased morbidity risk, whereas others found no differences between Hinchey III and IV patients [11, 22, 25, 30]. Nevertheless, despite the absence of results from larger cohorts of Hinchey IV, the majority of national and international guidelines still state the choice of PA with proximal diversion as a possible treatment option for these patients [5].

There are some limitations to the present study that are important to acknowledge. Most of the included studies consisted of small patient groups and were prone to selection bias due to their retrospective design. More importantly, there was substantial methodological heterogeneity between included studies, for both observational and randomized studies. For instance, differences in intraoperative details, follow-up duration, and definitions of morbidity were present. In order to reduce the effect of this heterogeneity, subgroup analyses of the included RCTs were performed, which for some outcomes showed differences with outcomes from observational studies. Nevertheless, even between these trials, several methodological differences existed, such as the moment of randomization, outcome definitions, and follow-up duration.

Interestingly, all four trials were terminated early for reasons of difficulties with patient accrual, which corresponds with the evidence that trials in the acute care setting are notoriously difficult to conduct and more often lead to early discontinuation [42]. However, more importantly, it should clearly be noted that these trial populations might still be a selected patient sample, as the decision to randomize an eligible patient might have been subject to surgeon’s preference. A comparison with eligible non-included patients could have helped objectify this potential bias and increase external generalizability, but was only reported in the Ladies trial. Additionally, the trial by Oberkofler and colleagues briefly reported on the numbers of patients that were not screened for eligibility or were not included after screening, but it did not compare patient and disease characteristics of these groups with those of the included patients [23]. Furthermore, high-risk patients (e.g., hemodynamically unstable or immunocompromised) were not included or underrepresented in this systematic review. For example, two of the four trials specifically stated hemodynamic instability to be an exclusion criterion. Hence, even though the evidence identifies PA to be the preferred approach to HP, accurate patient selection still remains key. Indeed, in a recent evidence-based EAES/SAGES consensus report, it was stated that PA with proximal diversion should be considered over HP in the appropriate clinical setting, but that HP remains the preferred operation for hemodynamically unstable patients [43].

To overcome some of the mentioned methodological problems and to find evidence to fill in the identified gaps in current knowledge, future research might benefit from gathering data in the context of multi-center or (inter)national audit studies. Through multi-center collaboration and prospective (preferably long term) data collection in a large sample of patients, such a study design has the ability to provide insights into current clinical practice and treatment trends, and to analyze outcomes with adjustment for known confounders, as well as to assess outcomes in subgroups such as PA patients with or without ileostomy or Hinchey IV patients. Moreover, the role of emergency laparoscopic sigmoidectomy could potentially be further assessed in this context, as recent promising evidence found it to be superior in terms of postoperative morbidity and hospital stay and concluded it to be feasible in selected patients and performed by experienced hands [32, 44]. The DAMASCUS study, a snapshot collaborative audit study on treatment of acute diverticulitis, is an example of such a design and its results are awaited with interest, https://www.thedukesclub.org.uk/wp-content/uploads/2019/07/DAMASCUS-Study-Summary.pdf. Lastly, with regard to rates of stoma reversal, Hartmann’s reversals in particular, it can be hypothesized that restoration of continuity will take place either later or not at all in those patients that have an impaired clinical condition. This is already partly reflected in the reported reasons for non-reversal within the published trials, but is also of great value to assess within the long-term follow-up of existing or novel studies.

In conclusion, this updated systematic review and meta-analysis provides several arguments to prefer PA over HP for the treatment of perforated diverticulitis with purulent or fecal peritonitis. Importantly, between-study heterogeneity needs to be considered while interpreting the present results and, above all, the findings should be interpreted within the context of hemodynamically stable and immunocompetent patients. In addition, this study identified gaps in current knowledge that are of interest for future investigation and of which results might further aid accurate surgical decision-making and optimal treatment within the setting of perforated diverticulitis.

## Electronic supplementary material

ESM 1 Appendix Hartmann’s procedure versus resection with primary anastomosis for perforated diverticulitis with purulent or fecal peritonitis: a systematic review and meta-analysis. (DOCX 13 kb)

ESM 2 Appendix - Tables Hartmann’s procedure versus resection with primary anastomosis for perforated diverticulitis with purulent or fecal peritonitis: a systematic review and meta-analysis. (DOCX 51 kb)

ESM 3 Figure S1 (PNG 1711 kb)

High Resolution Image (TIF 1899 kb)

ESM 4 Figure S2a (PNG 103 kb)

High Resolution Image (EPS 229 kb)

ESM 5 Figure S2b (PNG 103 kb)

High Resolution Image (EPS 227 kb)

ESM 6 Figure S2c (PNG 103 kb)

High Resolution Image (EPS 226 kb)

ESM 7 Figure S3a (PNG 104 kb)

High Resolution Image (EPS 233 kb)

ESM 8 Figure S3b (PNG 97 kb)

High Resolution Image (EPS 206 kb)

ESM 9 Figure S3c (PNG 104 kb)

High Resolution Image (EPS 228 kb)

ESM 10 Figure S3d (PNG 103 kb)

High Resolution Image (EPS 227 kb)

ESM 11 Figure S3e (PNG 99 kb)

High Resolution Image (EPS 217 kb)

ESM 12 Figure S3f (PNG 104 kb)

High Resolution Image (EPS 230 kb)

ESM 13 Figure S4a (PNG 111 kb)

High Resolution Image (EPS 250 kb)

ESM 14 Figure S4b (PNG 113 kb)

High Resolution Image (EPS 255 kb)

ESM 15 Figure S4c (PNG 112 kb)

High Resolution Image (EPS 248 kb)

## Data Availability

Not applicable.
