# Supplementary material for: Sigmoid resection with primary anastomosis versus the Hartmann’s procedure for perforated diverticulitis with purulent or fecal peritonitis: a systematic review and meta-analysis
Source: Int J Colorectal Dis. 2020 Jun 5;35(8):1371–86. doi: 10.1007/s00384-020-03617-8 (PMC7340681; doi:10.1007/s00384-020-03617-8)
Supplement: Supplementary file 1 — Appendix Hartmann’s procedure versus resection with primary anastomosis for perforated diverticulitis with purulent or fecal peritonitis: a systematic review and meta-analysis. (DOCX 13 kb) [file 384_2020_3617_MOESM1_ESM.docx]

**Appendix** Hartmann’s procedure versus resection with primary anastomosis for perforated diverticulitis with purulent or fecal peritonitis: a systematic review and meta-analysis

**Search syntaxes and results per database**

| embase.com | 1211 | 1199 |
| --- | --- | --- |
| Medline Ovid | 764 | 179 |
| Web of science | 569 | 176 |
| Cochrane CENTRAL | 34 | 6 |
| **Total** | **2578** | **1560** |

*embase.com*
('hartmann procedure'/de OR 'hartmann operation'/de OR (hartman* OR ((non-restor* OR nonrestor*) NEAR/3 resect*)):ab,ti OR (((('diverticulitis'/exp OR diverticulosis/exp) AND ('perforation'/exp OR 'acute disease'/de OR peritonitis/de)) OR ((diverticul* AND (perforat* OR complicat* OR acute OR peritonitis)) OR Hinchey*):ab,ti) AND ('colostomy'/exp OR 'colon stoma'/de OR (colostom* OR (colon* NEAR/3 stoma*) OR (staged NEAR/3 procedure*) OR two-stage* OR 2-stage*):ab,ti))) AND ('anastomosis'/de OR 'ileostomy'/de OR (((primar* OR end-to-end OR end-to-side OR side-to-end OR side-to-side) NEAR/3 anastomos*) OR ileostom*):ab,ti)

*Medline Ovid*
((hartman* OR ((non-restor* OR nonrestor*) ADJ3 resect*)).ab,ti. OR ((((exp Diverticulitis/ OR Diverticulum/ OR Diverticulosis, Colonic/) AND (exp Intestinal Perforation/ OR Acute Disease/ OR Peritonitis/)) OR ((diverticul* AND (perforat* OR complicat* OR acute OR peritonitis)) OR Hinchey*).ab,ti.) AND (colostomy/ OR (colostom* OR (colon* ADJ3 stoma*) OR (staged ADJ3 procedure*) OR two-stage* OR 2-stage*).ab,ti.))) AND (Anastomosis, Surgical/ OR Ileostomy/ OR (((primar* OR end-to-end OR end-to-side OR side-to-end OR side-to-side) ADJ3 anastomos*) OR ileostom*).ab,ti.)

*Web of science*
TS=(((hartman* OR (("non-restor*" OR nonrestor*) NEAR/2 resect*)) OR ((((diverticul* AND (perforat* OR complicat* OR acute OR peritonitis)) OR Hinchey*)) AND ((colostom* OR (colon* NEAR/2 stoma*) OR (staged NEAR/2 procedure*) OR two-stage* OR "2-stage*")))) AND ((((primar*) NEAR/2 anastomos*) OR ileostom*)))

*Cochrane CENTRAL*
((hartman* OR ((non next restor* OR nonrestor*) NEAR/3 resect*)):ab,ti OR ((((diverticul* AND (perforat* OR complicat* OR acute OR peritonitis)) OR Hinchey*):ab,ti) AND ((colostom* OR (colon* NEAR/3 stoma*) OR (staged NEAR/3 procedure*) OR two next stage* OR 2 next stage*):ab,ti))) AND ((((primar*) NEAR/3 anastomos*) OR ileostom*):ab,ti)
