# Supplementary material for: Sigmoid resection with primary anastomosis versus the Hartmann’s procedure for perforated diverticulitis with purulent or fecal peritonitis: a systematic review and meta-analysis
Source: Int J Colorectal Dis. 2020 Jun 5;35(8):1371–86. doi: 10.1007/s00384-020-03617-8 (PMC7340681; doi:10.1007/s00384-020-03617-8)
Supplement: Supplementary file 2 — Appendix - Tables Hartmann’s procedure versus resection with primary anastomosis for perforated diverticulitis with purulent or fecal peritonitis: a systematic review and meta-analysis. (DOCX 51 kb) [file 384_2020_3617_MOESM2_ESM.docx]

**Appendix - Tables** Hartmann’s procedure versus resection with primary anastomosis for perforated diverticulitis with purulent or fecal peritonitis: a systematic review and meta-analysis.

| **Supplemental Table 1** Operative characteristics of the index procedure | | | | | | | | | | | | | |
| --- | --- | --- | --- | --- | --- | --- | --- | --- | --- | --- | --- | --- | --- |
| **Study** | **Group** | **Patients (n)** | **Operating time (min)** | **Surgical expertise** | **Night surgery (n)** | **Blood loss (ml)** | **Open/laparoscopic** | **Conversion** | **Anastomotic configuration** | **Anastomotic construction** | **Stoma construction (n)** | **Drain placement** | **Intraoperative lavage** |
| **Randomized controlled trials** | | | | | | | | | | | | | |
| Binda | Overall | 90 | n.r. | n.r. | n.r. | n.r. | n.r. | n.r. | n.r. | n.r. | n.r. | Closed suction drainage along left paracolic gutter | Lavage of the peritoneal cavity with normal saline (0.9% NaCl) |
|  | HP | 56 | 154.4 (6.6) | n.r. | 36 (63.6) | n.r. | 53/3 (94.6/5.4) | 0 | n.r. | n.a. | 56 (100) | 52 (93) | 6.1 (0.6) liter |
|  | PA | 34 | 167.3 (8.5) | n.r. | 15 (44.1) | n.r. | 30/4 (88.2/11.8) | 0 | n.r. | Double-stapled anastomosis | 34 (100) | 32 (94) | 4.5 (0.5) liter |
| Bridoux | HP | 52 | 120 (40-360) | 23 (45.1%)* | 22 (43.1) | n.r. | n.r. | n.a. | n.r. | n.r. | 52 | Discretion of surgeon | Discretion of surgeon |
|  | PA | 50 | 175.5 (74-320) | 24 (48%)* | 19 (38) | n.r. | n.r. | n.a. | End-to-end or side-to-end | n.r. | 35 | Discretion of surgeon | Discretion of surgeon |
| Lambrichts | Overall | 130 | n.r. | - | n.r. | - | - | 0 | - | - | - | Decision left at the surgeon’s discretion | n.r. |
|  | HP | 66 | 118 (95.5-135.3) | 57 (86%)# | n.r. | ≤100: 30 (45%), 101-500: 24 (36%), 501-1000: 3 (5%), missing: 9 (14%) | 46/20 (70/30) | 0 | 1 (2%) STS | 1 (2%) manual | 65 (98%) | 21 (32%) | n.r. |
|  | PA | 64 | 125 (110-154) | 58 (91%)# | n.r. | ≤100: 31 (48%), 101-500: 20 (31%), 501-1000: 4 (6%), >1000: 1 (2%), missing: 8 (13%) | 47/17 (73/27) | 0 | 18 (28%) ETE, 3 (5%) ETS, 9 (14%) STS, 20 (31%) STE, missing 6 (9%) | 11 (17%) manual, 43 (67%) stapler, 2 (3%) missing | 17 (27%) | 27 (44%) | n.r. |
| Oberkofler | Overall | 62 | 180 (147-225) | 61/1 (98/2%)† | n.r. | 100 (200-575) | n.r. | n.a. | n.r. | n.r. | - | n.r. | Decided by individual surgeons |
|  | HP | 30 | 168 (130-220) | 31/1 (97/3%)† | n.r. | 300 (100-600) | n.r. | n.a. | n.r. | n.r. | 30 EC | n.r. | n.r. |
|  | PA | 32 | 208 (150-223) | 30/0 (100/0%)† | n.r. | 200 (100-500) | n.r. | n.a. | n.r. | Transanal circular stapling | 29 LI; 3 EC | n.r. | Colonic cleaning in 50% |
| **Observational studies** | | | | | | | | | | | | | |
| Gooszen | HP | 28 | 150 (60-240) | 28 (100%)§ | n.r. | n.r. | n.r. | n.a. | n.r. | n.r. | 28 (100) | n.r. | n.r. |
|  | PA | 32 | 172 (75-300) | 32 (100%)§ | n.r. | n.r. | n.r. | n.a. | n.r. | n.r. | 7 LI (21.9); 25 LTC (78.1) | n.r. | n.r. |
| Mueller | HP | 26 | n.r. | n.r. | n.r. | n.r. | n.r. | n.a. | n.r. | n.r. | Hinchey III: 9 EC Hinchey IV: 3 EC | n.r. | n.r. |
|  | PA | 47 | n.r. | n.r. | n.r. | n.r. | n.r. | n.a. | n.r. | n.r. | Hinchey III: 2 LI  Hinchey IV: n.r. | n.r. | n.r. |
| Regenet | Overall | 60 | n.r. | Five surgeons with the same level of training participated | n.r. | n.r. | 60/0 (100/0) | n.a. | n.a. | n.r. | - | Pouch of Douglas was always drained | Peritoneal lavage in all, with warm saline (mean 10 l (range 7-120)) |
|  | HP | 33 | 172 (22) | - | n.r. | n.r. | 33/0 (100/0) | n.a. | n.a. | n.r. | 33 EC | - | Irrigation of colon and rectal stump with saline (37C) |
|  | PA | 27 | 225 (39) | - | n.r. | n.r. | 27/0 (100/0) | n.a. | STE in all patients | n.r. | 27 LI | - | - |
| Richter | Overall | 41 | 131.4 (5) | Staff surgeons (8), certified surgeons (5), residents under staff supervision (3) | n.r. | 221 (35) | n.r. | n.a. | n.r. | n.r. | - | n.r. | Abdominal lavage with 30 liter of warm Ringer's lactate solution |
|  | HP | 5 | n.r. | - | n.r. | - | n.r. | n.a. | n.r. | n.r. | 5 EC | n.r. | - |
|  | PA | 36 | n.r. | - | n.r. | - | n.r. | n.a. | n.r. | n.r. | 4 LI | n.r. | - |
| Schilling | HP | 42 | 198 (60) | n.r. | n.r. | n.r. | n.r. | n.a. | n.r. | n.r. | 42 EC | n.r. | Extensive abdominal lavage with at least 20 liter warm (37°C) Ringer's lactate |
|  | PA | 13 | 198 (72) | n.r. | n.r. | n.r. | n.r. | n.a. | n.r. | Two-layer technique with absorbable suture | 13 LI | n.r. | Extensive abdominal lavage with at least 20 liter warm (37°C) Ringer's lactate |
| Thaler | HP | 62 | n.r. | All procedures performed by eight experienced staff surgeons | n.r. | n.r. | n.r. | n.a. | n.r. | n.r. | 62 EC | n.r. | n.r. |
|  | PA | 20 | n.r. | All procedures performed by eight experienced staff surgeons | n.r. | n.r. | n.r. | n.a. | n.r. | n.r. | 0 | n.r. | n.r. |
| Trenti | HP | 60 | n.r. | 6 CS (27.3%), 23 (67.6%)● | n.r. | n.r. | n.r. | n.a. | n.r. | n.r. | 60 EC | n.r. | Extensive intraabdominal lavage with warm saline solution |
|  | PA | 27 | n.r. | 16 CS (72.7%), 11 (32.4%)● | n.r. | n.r. | n.r. | n.a. | n.r. | n.r. | 5 LI | n.r. | Extensive intraabdominal lavage with warm saline solution |
| Vennix | PM (open) | 78 | 96.5 (87-120) | 76 (97.4%)† | n.r. | 15 (42) | 78 | n.r. | n.r. | n.r. | 12/27 (44.4%) | n.r. | n.r. |
|  | PM (laparoscopic) | 39 | 127 (105-159) | 38 (97.4%)† | n.r. | 14 (74) | 39 | Overall (laparoscopic): 51/153 (33.3%) | n.r. | n.r. | 8/13 (61.5%) | n.r. | n.r. |
|  | HP | n.r. | n.r. | n.r. | n.r. | n.r. | n.r. | n.r. | n.r. | n.r. | n.r. | n.r. | n.r. |
|  | PA | n.r. | n.r. | n.r. | n.r. | n.r. | n.r. | n.r. | n.r. | n.r. | n.r. | n.r. | n.r. |
| Vermeulen 2007 | HP | 139 | n.r. | 89 GS (64%), 50 CS (36%) | 89 DOH (64%), 50 OOH (36%) | n.r. | n.r. | n.a. | n.r. | n.r. | 139 EC | n.r. | n.r. |
|  | PA | 61 | n.r. | 38 GS (62%), 23 CS (38%) | 27 DOH (45%), 34 OOH (55%) | n.r. | n.r. | n.a. | n.r. | n.r. | 16 (26%) LI | n.r. | n.r. |
| Wright | Overall (colorectal) | 62 | 154 (30-482) | 22 PGY3-4 (35.5%), 40 PGY5-6 (64.5%) | n.r. | n.r. | 10 (16.1) | 2 (20) | n.r. | n.r. | - | n.r. | n.r. |
|  | Overall (general) | 53 | 127 (39-239) | 26 PGY3-4 (49.1%), 27 PGY5-6 (50.9%) | n.r. | n.r. | 5 (9.4) | 2 (40) | n.r. | n.r. | - | n.r. | n.r. |
|  | HP (colorectal) | 21 | 137 | n.a. | n.r. | n.r. | - | - | n.r. | n.r. | 21 EC | n.r. | n.r. |
|  | HP (general) | 34 | 128 | n.a. | n.r. | n.r. | - | - | n.r. | n.r. | 34 EC | n.r. | n.r. |
|  | PA (colorectal) | 38 | n.r. | n.a. | n.r. | n.r. | - | - | n.r. | n.r. | 28 LI | n.r. | n.r. |
|  | PA (general) | 15 | n.r. | n.a. | n.r. | n.r. | - | - | n.r. | n.r. | 0 LI | n.r. | n.r. |
| Continuous data are median (interquartile range), mean (standard deviation), or mean (range). *Resident as first surgeon. §In all cases, the operation was performed by two surgeons of whom at least one had colorectal expertise. #Gastrointestinal surgeon present. †Presence of board-certified surgeon. ●Calculated including only patients with a peritonitis severity score <11. CS, colorectal surgeon; DOH, during office hours; EC, end colostomy; ETE, end-to-end anastomosis; ETS, end-to-side anastomosis; GS, general surgeon; HP, Hartmann’s procedure; LI, loop ileostomy; LTC, loop transverse colostomy; n.a., not applicable; n.r., not reported; OOH, outside office hours; PA, primary anastomosis; PGY, postgraduate year. PM, propensity-matched cohort; STE, side-to-end anastomosis; STS, side-to-side anastomosis. | | | | | | | | | | | | | |

| **Supplemental Table 2** Operative characteristics of the reversal procedure | | | | | | | | | | | |
| --- | --- | --- | --- | --- | --- | --- | --- | --- | --- | --- | --- |
| **Study** | **Group** | **Patients (n)** | **Operating time (min)** | **Surgical expertise** | **Blood loss (ml)** | **Open/laparoscopic** | **Conversion** | **Anastomotic configuration** | **Anastomotic construction** | **Drain placement** | **Intraoperative lavage** |
| **Randomized controlled trials** | | | | | | | | | | | |
| Binda | HP | 34 | n.r. | n.r. | n.r. | Open | n.a. | n.r. | Linear stapler | n.r. | n.r. |
|  | PA | 22 | n.r. | n.r. | n.r. | Performed with trephine incision | n.a. | Functional ETE | Cutting stapler | n.r. | n.r. |
| Bridoux | Overall | 65 | 120 (30-510) | 29 (44.6%)* | n.r. | n.r. | n.a. | n.r. | n.r. | n.r | n.r. |
|  | HP | 33 | 170 (80-510) | 13 (39.4%)* | n.r. | n.r. | n.a. | n.r. | n.r. | n.r | n.r. |
|  | PA | 32 | 70 (30-300) | 16 (50%)* | n.r. | n.r. | n.a. | n.r. | n.r. | n.r | n.r. |
| Lambrichts | HP | 44 | n.r. | n.r. | n.r. | 24/20 | n.r. | n.r. | n.r. | n.r. | n.r. |
|  | PA | 38 | n.r. | n.r. | n.r. | Ileostomy reversal: 34, colostomy reversal: 3/1 | n.r. | n.r. | n.r. | n.r. | n.r. |
| Oberkofler | Overall | 41 | 110 (66-158) | n.r. | 25 (5-50) | n.r. | n.a. | n.r. | n.r. | n.r. | n.r. |
|  | HP | 15 | 183 (150-225) | n.r. | 45 (5-150) | n.r. | n.a. | n.r. | n.r. | n.r. | n.r. |
|  | PA | 26 | 73 (60-90) | n.r. | 20 (5-40) | n.r. | n.a. | n.r. | n.r. | n.r. | n.r. |
| **Observational studies** | | | | | | | | | | | |
| Gooszen | HP | 13 | 172 (90-195) | n.r. | n.r. | n.r. | n.a. | n.r. | n.r. | n.r. | n.r. |
|  | PA | 24 | 75 (30-150) | n.r. | n.r. | n.r. | n.a. | n.r. | n.r. | n.r. | n.r. |
| Wright | Overall (colorectal) | 38 | 78 (33-406) | n.a. | n.r. | n.r. | n.a. | n.r. | n.r. | n.r. | n.r. |
|  | Overall (general) | 22 | 167 (85-473) | n.a. | n.r. | n.r. | n.a. | n.r. | n.r. | n.r. | n.r. |
| Continuous data are median (interquartile range), mean (standard deviation), or mean (range). *Resident as first surgeon. CS, colorectal surgeon; ETE, end-to-end anastomosis; HP, Hartmann’s procedure; n.a., not applicable; n.r., not reported; NCS, non-colorectal surgeon; PA, primary anastomosis; STE, side-to-end anastomosis; STS, side-to-side anastomosis. | | | | | | | | | | | |

| **Supplemental Table 3** Summarized outcomes of quantitative analyses of the baseline characteristics in observational studies | | | | | | | |
| --- | --- | --- | --- | --- | --- | --- | --- |
| **Characteristic** | **Pooled outcome*** | **95% CI** | **P value** | **I^2^ (%)** | **Chi^2^** | **P value** | **Studies (n)** |
| Sex (% female) | 2.14 | 0.79, 5.75 | 0.13 | 54 | 6.47 | 0.09 | 4 |
| Mean age (years) | -4.84 | -9.41, -0.27 | 0.04 | 39 | 4.93 | 0.18 | 4 |
| ASA I-II (%) | 3.92 | 0.21, 72.52 | 0.36 | 93 | 14.29 | 0.0002 | 2 |
| Hinchey III (%) | 2.45 | 1.30, 4.63 | 0.006 | 0 | 3.53 | 0.62 | 6 |
| Mean MPI | -3.58 | -5.70, -1.47 | 0.0009 | 0 | 1.08 | 0.58 | 3 |
| Mean CRP | -29.58 | -67.74, 8.58 | 0.13 | 0 | 0.49 | 0.48 | 2 |
| *Pooled outcomes were OR for dichotomous variables and MD for continuous variables. Quantitative analysis was not possible for BMI, previous diverticulitis, previous abdominal surgery, and white blood cell count, as data was insufficient for these characteristics. | | | | | | | |

| **Supplemental Table 4** Details of randomized controlled trials | | | | | | | | | | | |
| --- | --- | --- | --- | --- | --- | --- | --- | --- | --- | --- | --- |
| **Study** | **Inclusion criteria** | **Exclusion criteria** | **Total sample size (n)** | **Patients screened for eligibility (n)** | **Patients included in (modified) intention-to-treat analyses (n)** | | **Cross-overs** | | **Moment of randomization** | **Primary endpoint** | **Trial accrual** |
|  |  |  |  |  | **HP** | **PA** | **HP** | **PA** |  |  |  |
| Binda | - ≥18 years of age  - perforated left colonic diverticulitis with peritonitis | - failure to sign consent  - peritonitis secondary to perforated diverticulitis of right colon | 600 | n.r. | 56 | 34 | None | None | Preoperative | n.r. | Early termination, because of slow patient accrual. |
| Bridoux | - ≥18 years of age  - perforated diverticulitis with purulent or fecal peritonitis | - physical states that prevented patient’s participation (e.g. septic shock or multivisceral failure)  - failure to provide consent | 246 | n.r. | - Primary analysis: 52  - Stoma reversal analysis: 33 | - Primary analysis: 50  - Stoma reversal analysis: 32 | - 1 total coloproctectomy | - 5 cross-overs to HP | Preoperative | Rate of mortality after index and reversal operation. | Early termination, because of recruitment difficulties. |
| Lambrichts | - patients 18-85 years of age  - clinical suspicion of perforated diverticulitis with peritonitis and free air/fluid on abdominal radiography or CT  - signed informed consent | - dementia  - previous sigmoidectomy  - previous pelvic radiotherapy  - chronic steroid treatment (≥ 20 mg daily)  - preoperative shock requiring inotropic support | 236 | n.r. | - Primary analysis: 66  - Stoma reversal analysis: 44 | - Primary analysis: 64  - Stoma reversal analysis: 38 | - 1 cross-over to PA | - 7 cross-overs to HP  - 1 cross-over to LL | Intraoperative | 12-month stoma-free survival. | Early termination, because of slow patient accrual. |
| Oberkofler | - ≥18 years of age  - perforated left colonic diverticulitis with peritonitis  - informed consent | - patients without generalized peritonitis (Hinchey I and II)  - evidence of metastasis at presentation | 136 | 83 (14 did not meet inclusion criteria, 7 declined to participate) | - Primary analysis: 30  - Stoma reversal analysis: 15 | - Primary analysis: 32  - Stoma reversal analysis: 26 | - 1 cross-over to PA | - 3 cross-overs to HP | Preoperative | Overall postoperative complication rate (percent yes/no) including the first (colon resection) and second (stoma reversal) operation, assessed according to the Clavien-Dindo classification. | Early termination, because of decreasing accrual rates and an interim analysis showing significant differences of relevant secondary endpoints. |
| HP, Hartmann’s procedure; LL, laparoscopic lavage; n.r., not reported; PA, primary anastomosis. | | | | | | | | | | | |

| **Supplemental Table 5** Numbers needed to treat | | | | |
| --- | --- | --- | --- | --- |
| **Outcomes** | **Risk difference (95% CI)** | **Risk Ratio (95% CI)** | **Assumed control risk** | **Numbers needed to treat*** |
| Reversal rate constructed stomas | 0.20 (0.08, 0.31) | 1.30 (1.12, 1.52) | 0.62 (126/203) | 5 |
| Number of stoma-free patients | 0.22 (0.10, 0.33) | 1.34 (1.16, 1.55) | 0.62 (127/204) | 5 |
| Overall morbidity after reversal procedure | -0.17 (-0.26, -0.08) | 0.44 (0.18, 1.07) | 0.27 (34/126) | 7 |
| *Numbers needed to treat were calculated as 1/\|assumed control risk*(1-risk ratio)\|, for which the assumed control risk was the pooled event rate in the HP group and outcomes were rounded to the nearest whole number. | | | | |

| **Supplemental Table 6a** Outcomes of the reversal procedure (1/2) | | | | | | | | | |
| --- | --- | --- | --- | --- | --- | --- | --- | --- | --- |
| **Study** | **Group** | **No. of stomas reversed/constructed (%)** | **Time interval to reversals** | **Mortality** | **Morbidity** | **Clavien-Dindo** | **Reoperations** | **LOS (days)** | **ICU stay** |
| **Randomized controlled trials** | | | | | | | | | |
| Binda | HP | 34/56 (60.7%) | 183.5 (121) days | 0 | 8 (23.5%)^a^ | n.r. | 3 (8.8%) | n.r. | n.r. |
|  | PA | 22/34 (64.7%) | 161.4 (141) days | 0 | 1 (4.5%)^a^ | n.r. | 1 (4.5%) | n.r. | n.r. |
| Bridoux | HP | 33/52 (63.5%) | n.r. | 1 (3%) | 7 (21.2%)^a^ | 3 (9%)^b^ | n.r. | 7 (3-22) | n.r. |
|  | PA | 32/35 (91.4%) | n.r. | 0 | 4 (12.5%)^a^ | 1 (3%)^b^ | n.r. | 5 (3-33) | n.r. |
| Lambrichts | HP | 44/65 (66.7%) | 133 (102-208) days | 0 | Major^c^: 7 (16%) Minor^c^: 6 (14%)  Overall^c^: 13 (30%) | n.r. | 4 (9%) | 5 (4-6) | n.r. |
|  | PA | 38/46 (82.6%) | 113.5 (80-155) days | 0 | Major^c^: 1 (3%) Minor^c^: 2 (5%)  Overall^c^: 3 (8%) | n.r. | 1 (3%) | 4 (2.8-5) | n.r. |
| Oberkofler | HP | 15/30 (50%) | Median: 6 months | 0 | 6 (40%)^d^ | 3 (20%)^e^ | n.r. | 9 (6-17) | n.r. |
|  | PA | 26/32 (81.3%) | Median: 3 months | 0 | 6 (23%)^d^ | 0^e^ | n.r. | 6 (4-10) | n.r. |
| **Observational studies** | | | | | | | | | |
| Regenet | HP | 20/29 (70%) | 151 (71.7) days | 0 | 5 (24%)^a^ | n.r. | n.r. | n.r. | n.r. |
|  | PA | 0/0 | n.a. | n.r. | n.r. | n.r. | n.r. | n.r. | n.r. |
| Richter | HP | 1/5 (20%) | 16 months | n.r. | n.r. | n.r. | n.r. | n.r. | n.r. |
|  | PA | n.r. | n.r. | n.r. | n.r. | n.r. | n.r. | n.r. | n.r. |
| Schilling | HP | 32/42 (76.2%) | 169 (74) days | n.r. | n.r. | n.r. | n.r. | 15.4 (8.4) | 0.8 (1.5) |
|  | PA | 0/0 | n.a. | n.r. | n.r. | n.r. | n.r. | n.a. | n.a. |
| Trenti | HP | 9/33 (27.3%) | 76.2 (63) days | n.r. | n.r. | n.r. | n.r. | n.r. | n.r. |
|  | PA | 3/5 (60%) | n.r. | n.r. | n.r. | n.r. | n.r. | n.r. | n.r. |
| Vennix | HP (open) | 0.64 12-month stoma-free probability | n.a. | n.r. | n.r. | n.r. | n.r. | n.r. | n.r. |
|  | HP (laparoscopic) | 0.88 12-month stoma-free probability | n.a. | n.r. | n.r. | n.r. | n.r. | n.r. | n.r. |
|  | PA (open) | 1.00 12-month stoma-free probability | n.a. | n.r. | n.r. | n.r. | n.r. | n.r. | n.r. |
|  | PA (laparoscopic) | 1.00 12-month stoma-free probability | n.a. | n.r. | n.r. | n.r. | n.r. | n.r. | n.r. |
| Wright | Overall (colorectal) | 20/25 (80%) | n.r. | n.r. | n.r.s. | n.r. | n.r. | n.r.s. | n.r. |
|  | Overall (general) | 20/30 (66.7%) | n.r. | n.r. | n.r.s. | n.r. | n.r. | n.r.s. | n.r. |
|  | HP (colorectal) | 10/21 (47.6%) | n.r.s. | n.r. | n.r.s. | n.r. | n.r. | n.r.s. | n.r. |
|  | HP (general) | 22/34 (64.7%) | n.r.s. | n.r. | n.r.s. | n.r. | n.r. | n.r.s. | n.r. |
|  | PA (colorectal) | n.r.s. | n.r.s. | n.r. | n.r.s. | n.r. | n.r. | n.r.s. | n.r. |
|  | PA (colorectal) | n.r.s. | n.r.s. | n.r. | n.r.s. | n.r. | n.r. | n.r.s. | n.r. |
| Continuous data are median (interquartile range), mean (standard deviation), or mean (range). a = overall morbidity; b = Clavien-Dindo III-V; c = major morbidity defined as surgical reintervention, percutaneous abscess drainage, fascial dehiscence, urosepsis, myocardial infarction, renal failure, and respiratory insufficiency, minor morbidity defined as surgical site infection, postoperative ileus, pneumonia, delirium, urinary tract infection, abscess without drainage, thrombosis, cardiac complications, and overall morbidity defined as major and minor complications combined; d = Clavien-Dindo I-V; e = Clavien-Dindo III-IV, serious complications. HP, Hartmann’s procedure; ICU, intensive care unit; LOS, length of stay; n.a., not applicable; n.r., not reported; n.r.s., not reported separately; | | | | | | | | | |

| **Supplemental Table 6b** Outcomes of the reversal operation (2/2) | | | | | | | | | | | | |
| --- | --- | --- | --- | --- | --- | --- | --- | --- | --- | --- | --- | --- |
| **Study** | **Group** | **Reversal (n)** | **Sepsis** | **Anastomotic leakage** | **Intra-abdominal abscess** | **Abscess drainage** | **SSI** | **Other infectious complications** | **Organ dysfunction** | **Fascial dehiscence** | **Incisional hernia** | **Stoma site incisional hernia** |
| **Randomized controlled trials** | | | | | | | | | | | | |
| Binda | HP | 34 | n.r. | 2 (5.9%) | 1 (2.9%) | 1 (2.9%) | 3 (8.8%) deep, 5 (14.7%) superficial | n.r. | n.r. | n.r. | n.r. | n.r. |
|  | PA | 22 | n.r. | 1 (4.5%) | 0 | 0 | 0 | n.r. | n.r. | n.r. | n.r. | n.r. |
| Bridoux | HP | 33 | n.r. | 0 | 1 (3%) | 1 (3%) | n.r. | n.r. | n.r. | n.r. | n.r. | n.r. |
|  | PA | 32 | n.r. | 1 (3%) | 0 | 0 | n.r. | n.r. | n.r. | n.r. | n.r. | n.r. |
| Lambrichts | HP | 44 | n.r. | 1 (2%) | 3 (7%) | 3 (7%) | 5 (11%) | 1 (2%) UTI | 0 | 1 (2%) | n.r | 2 (5%) |
|  | PA | 38 | n.r. | 0 | 0 | 0 | 1 (3%) | 0 | 0 | 0 | n.r. | 1 (3%) |
| Oberkofler | HP | 15 | 1 (6.7%) | 2 (13.3%) | n.r. | n.r. | 3 (20%) | n.r. | n.r. | n.r. | n.r. | n.r. |
|  | PA | 26 | 0 | 0 | n.r. | n.r. | 3 (11.5%) | n.r. | n.r. | n.r. | n.r. | n.r. |
| **Observational studies** | | | | | | | | | | | | |
| Trenti | HP | 9 | n.r. | 0 | n.r. | n.r. | n.r. | n.r. | n.r. | n.r. | n.r. | n.r. |
|  | PA | 3 | n.r. | 0 | n.r. | n.r. | n.r. | n.r. | n.r. | n.r. | n.r. | n.r. |
| Continuous data are median (interquartile range), mean (standard deviation), or mean (range). a = Complications concerning anastomotic healing, including leakage and anastomotic or presacral abscess. HP, Hartmann’s procedure; n.a., not applicable; n.r., not reported; n.r.s., not reported separately; PA, primary anastomosis; SSI, surgical site infection; UTI, urinary tract infection. | | | | | | | | | | | | |
