# Supplementary figures and images for: Sigmoid resection with primary anastomosis versus the Hartmann’s procedure for perforated diverticulitis with purulent or fecal peritonitis: a systematic review and meta-analysis
Source: Int J Colorectal Dis. 2020 Jun 5;35(8):1371–86. doi: 10.1007/s00384-020-03617-8 (PMC7340681; doi:10.1007/s00384-020-03617-8)

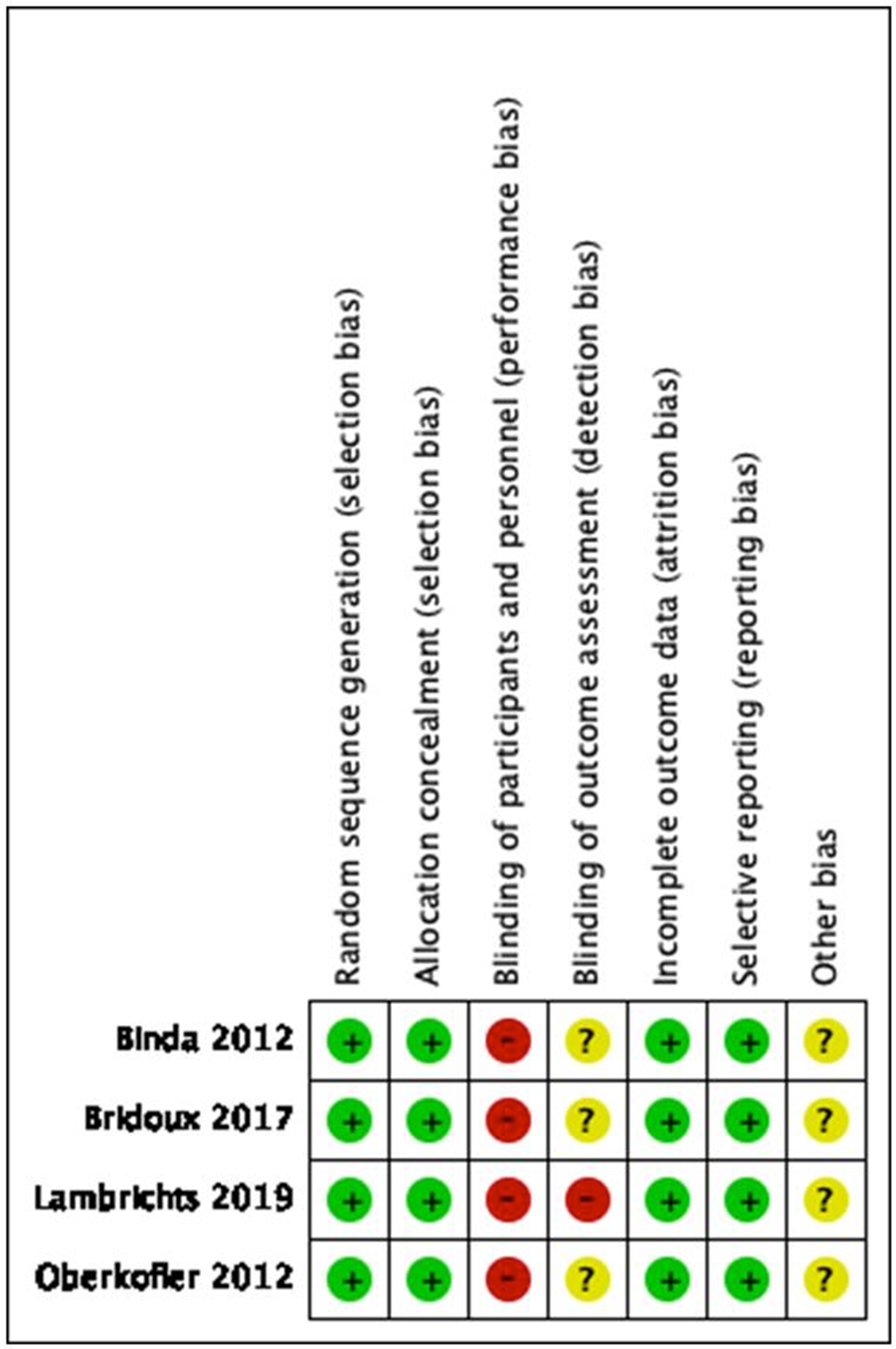

Supplement: Supplementary file 3 — Figure S1 (PNG 1711 kb) [file 384_2020_3617_Fig5_ESM.png]

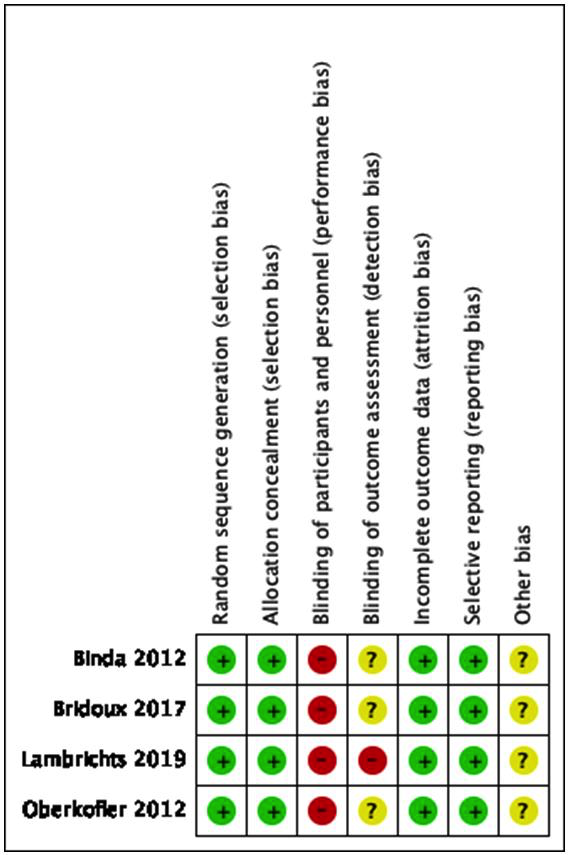

Supplement: Supplementary file 4 — High Resolution Image (TIF 1899 kb) [file 384_2020_3617_MOESM3_ESM.tif]

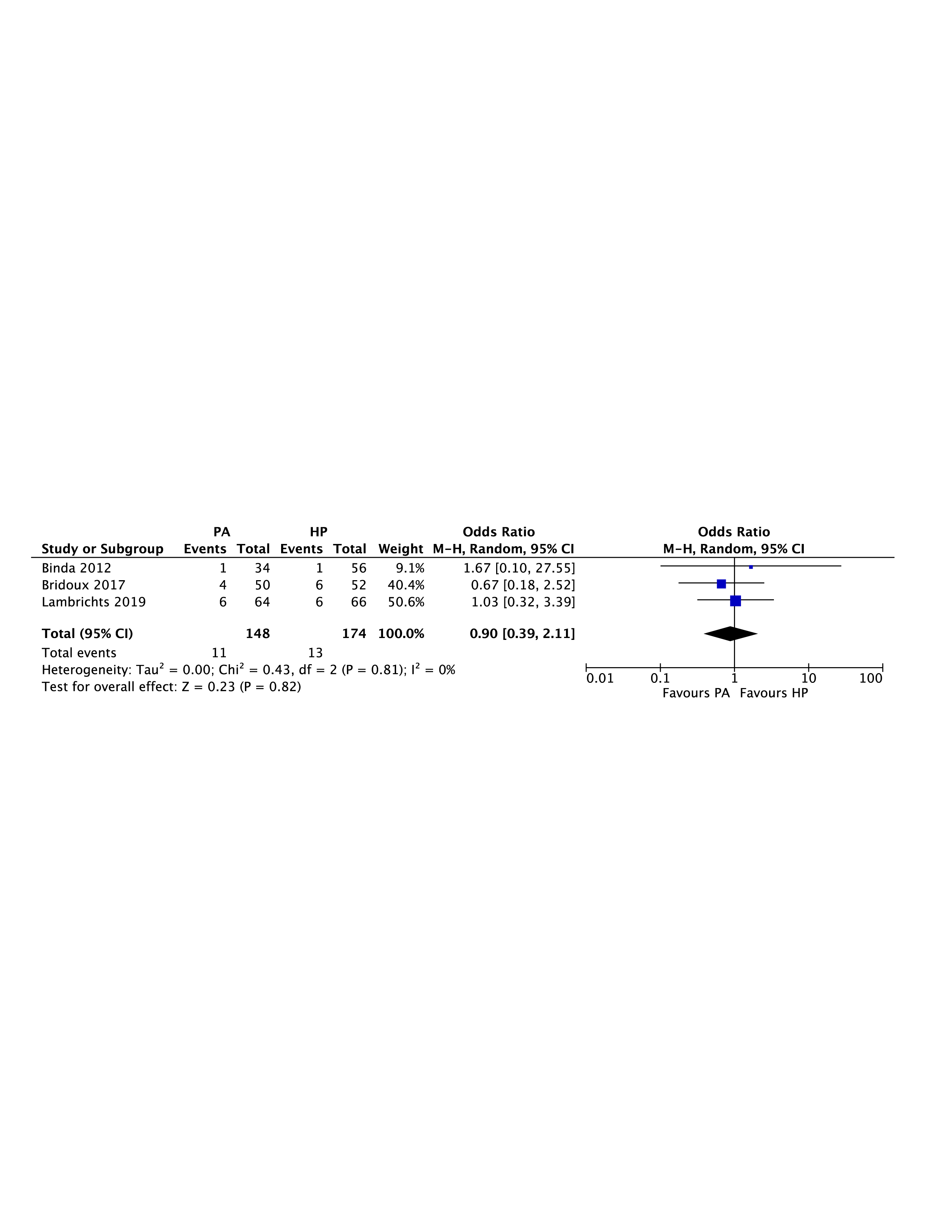

Supplement: Supplementary file 5 — Figure S2a (PNG 103 kb) [file 384_2020_3617_Fig6_ESM.png]

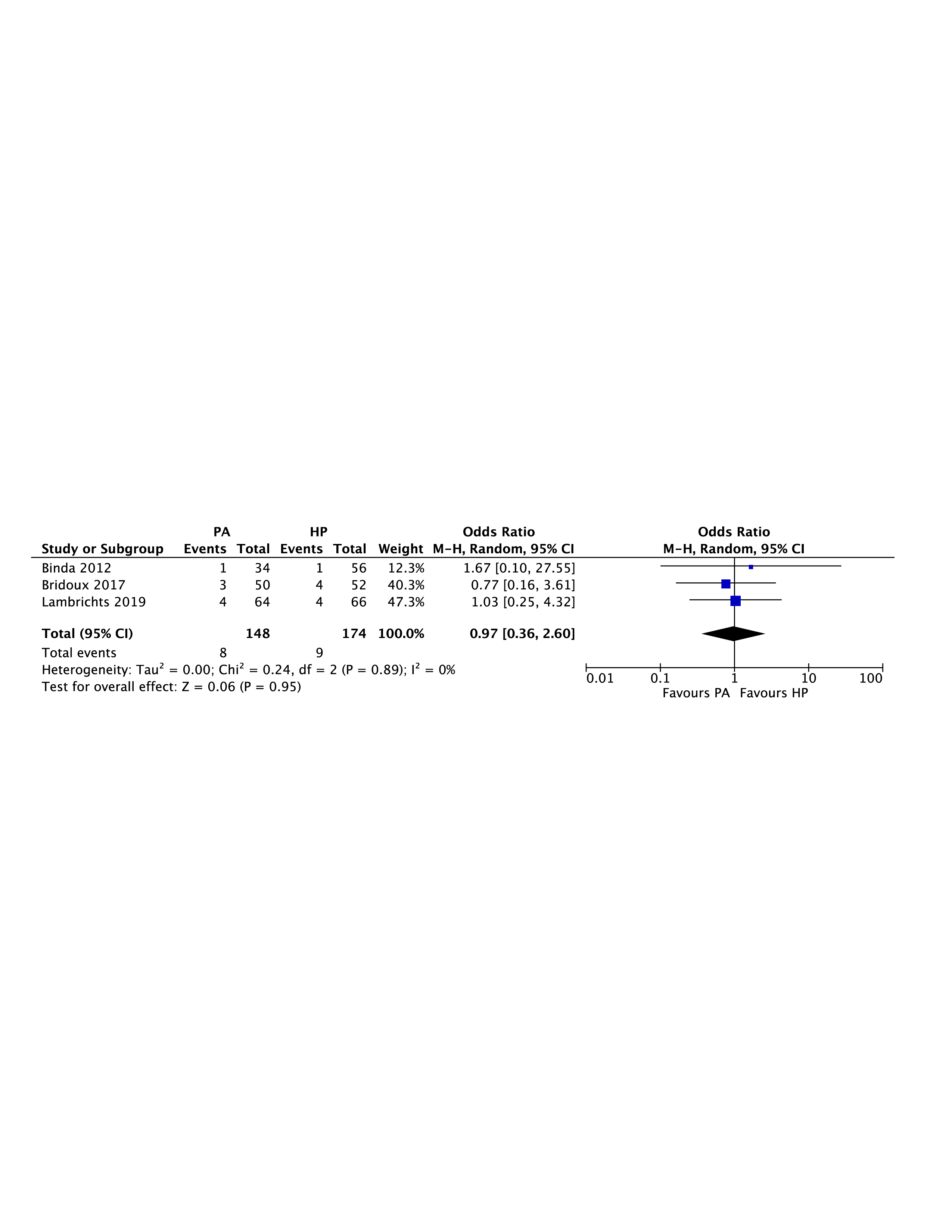

Supplement: Supplementary file 7 — Figure S2b (PNG 103 kb) [file 384_2020_3617_Fig7_ESM.png]

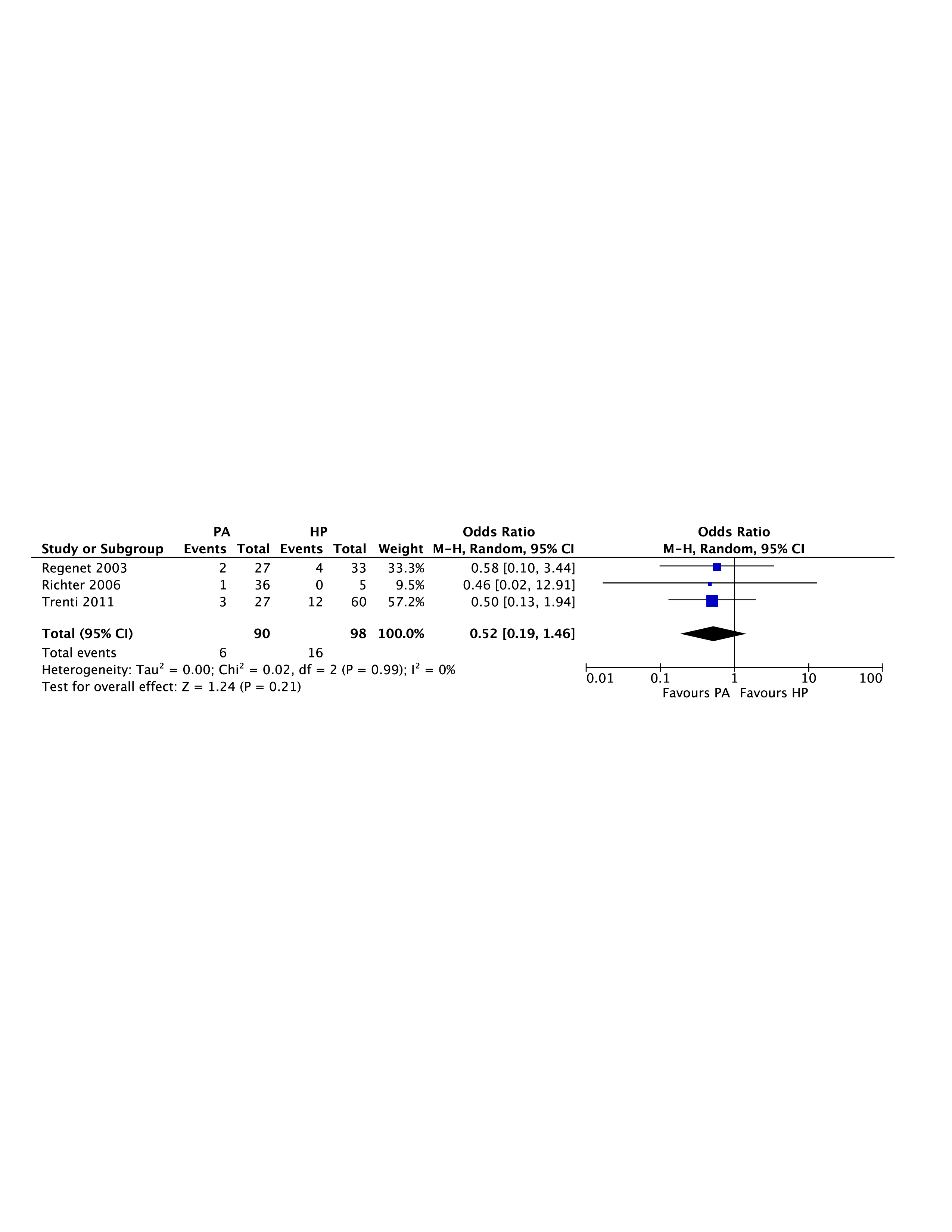

Supplement: Supplementary file 9 — Figure S2c (PNG 103 kb) [file 384_2020_3617_Fig8_ESM.png]

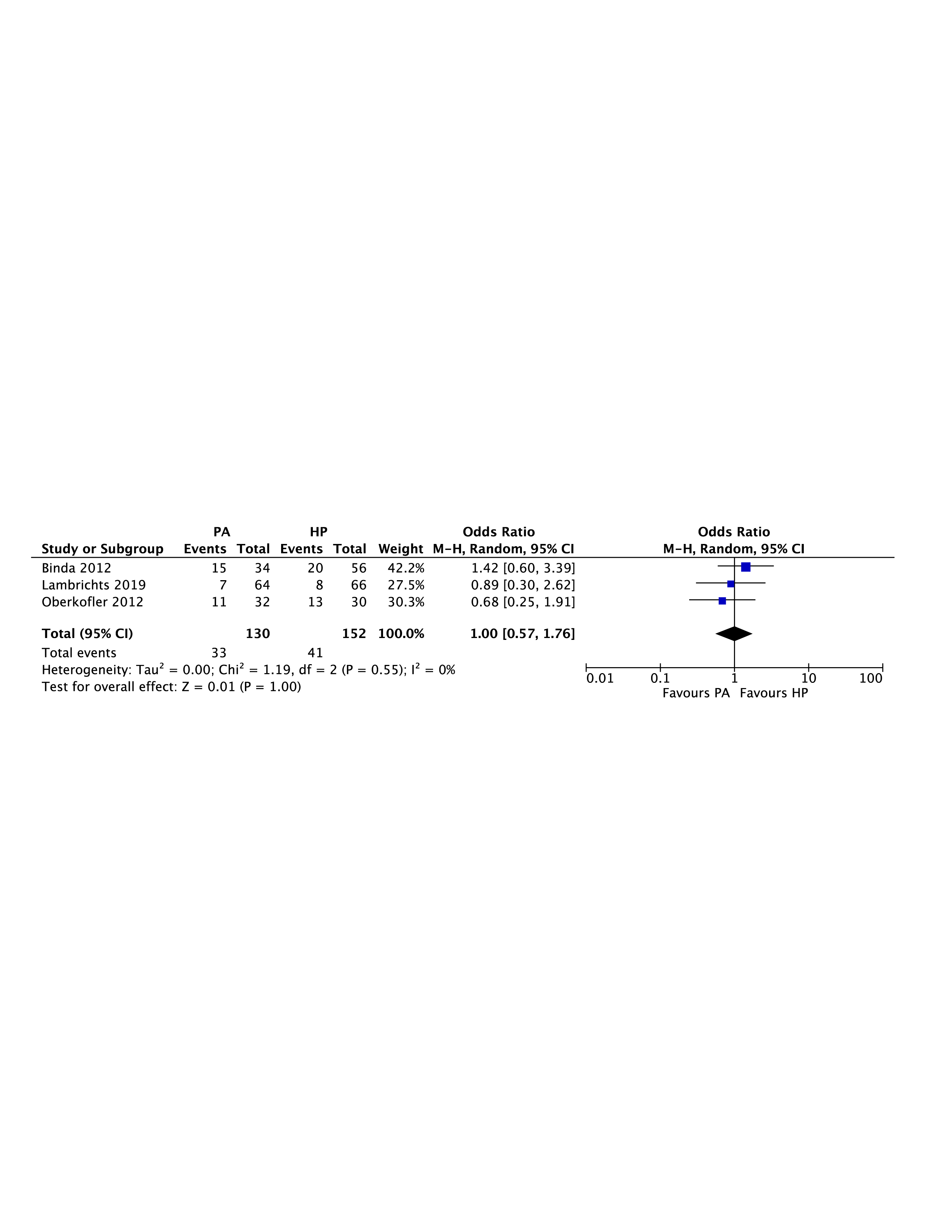

Supplement: Supplementary file 11 — Figure S3a (PNG 104 kb) [file 384_2020_3617_Fig9_ESM.png]

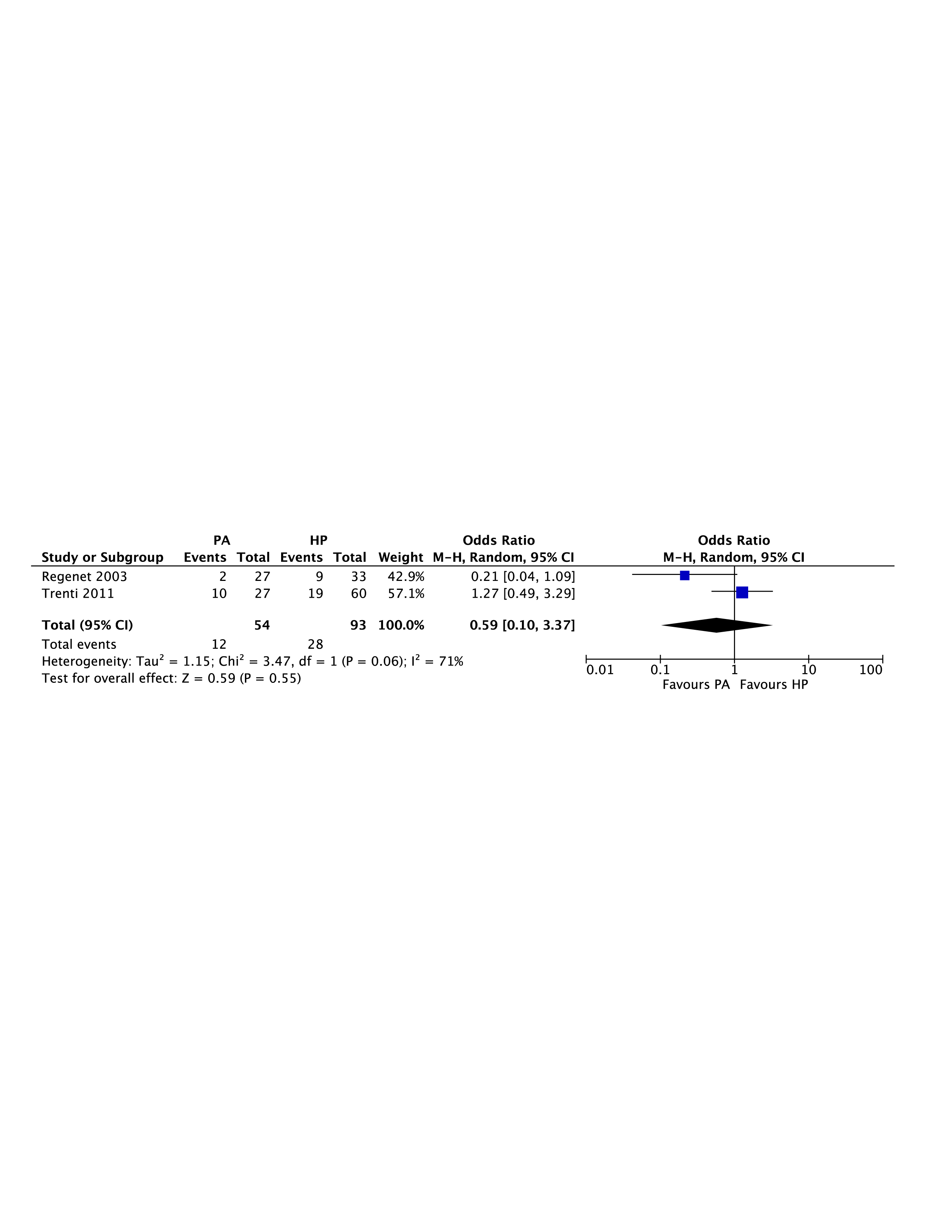

Supplement: Supplementary file 13 — Figure S3b (PNG 97 kb) [file 384_2020_3617_Fig10_ESM.png]

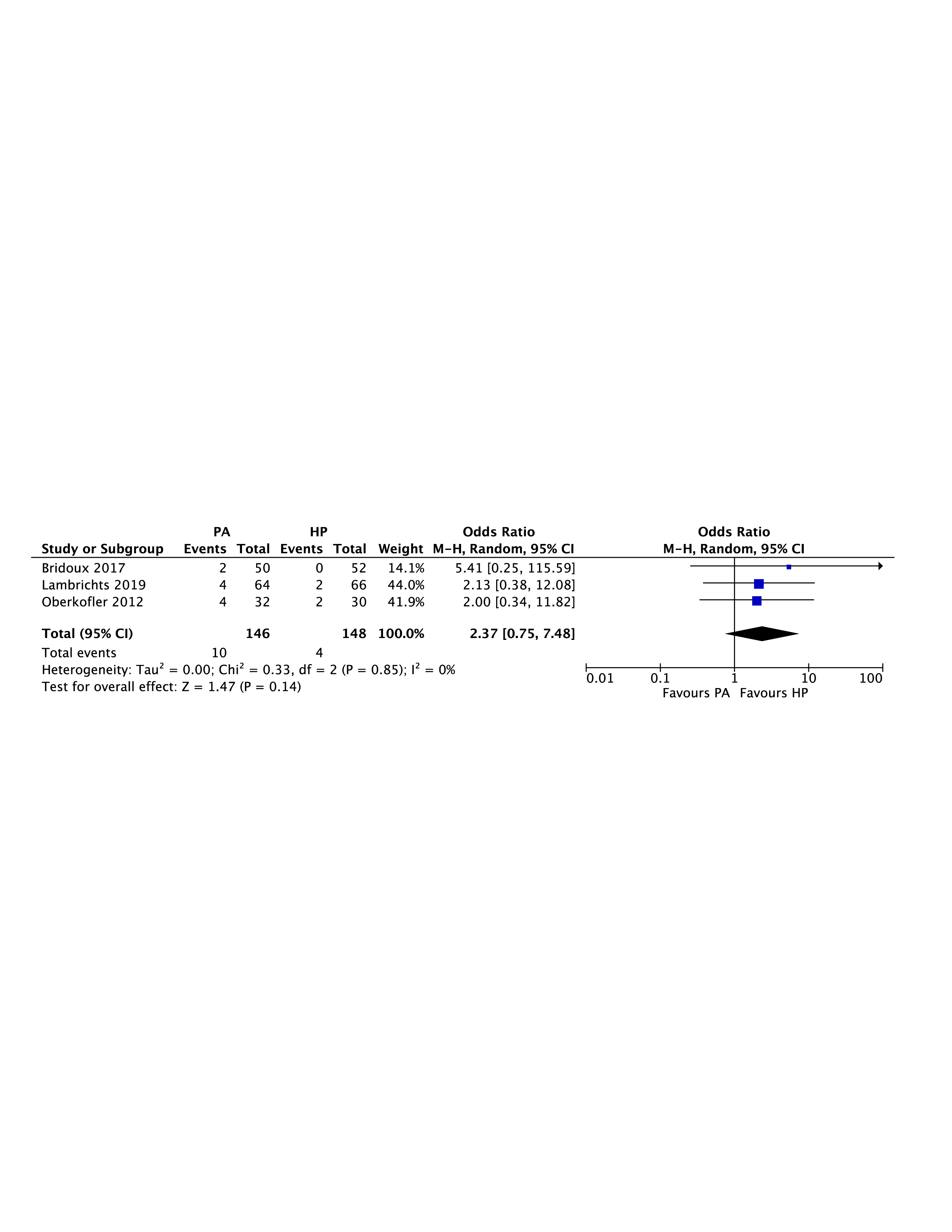

Supplement: Supplementary file 15 — Figure S3c (PNG 104 kb) [file 384_2020_3617_Fig11_ESM.png]

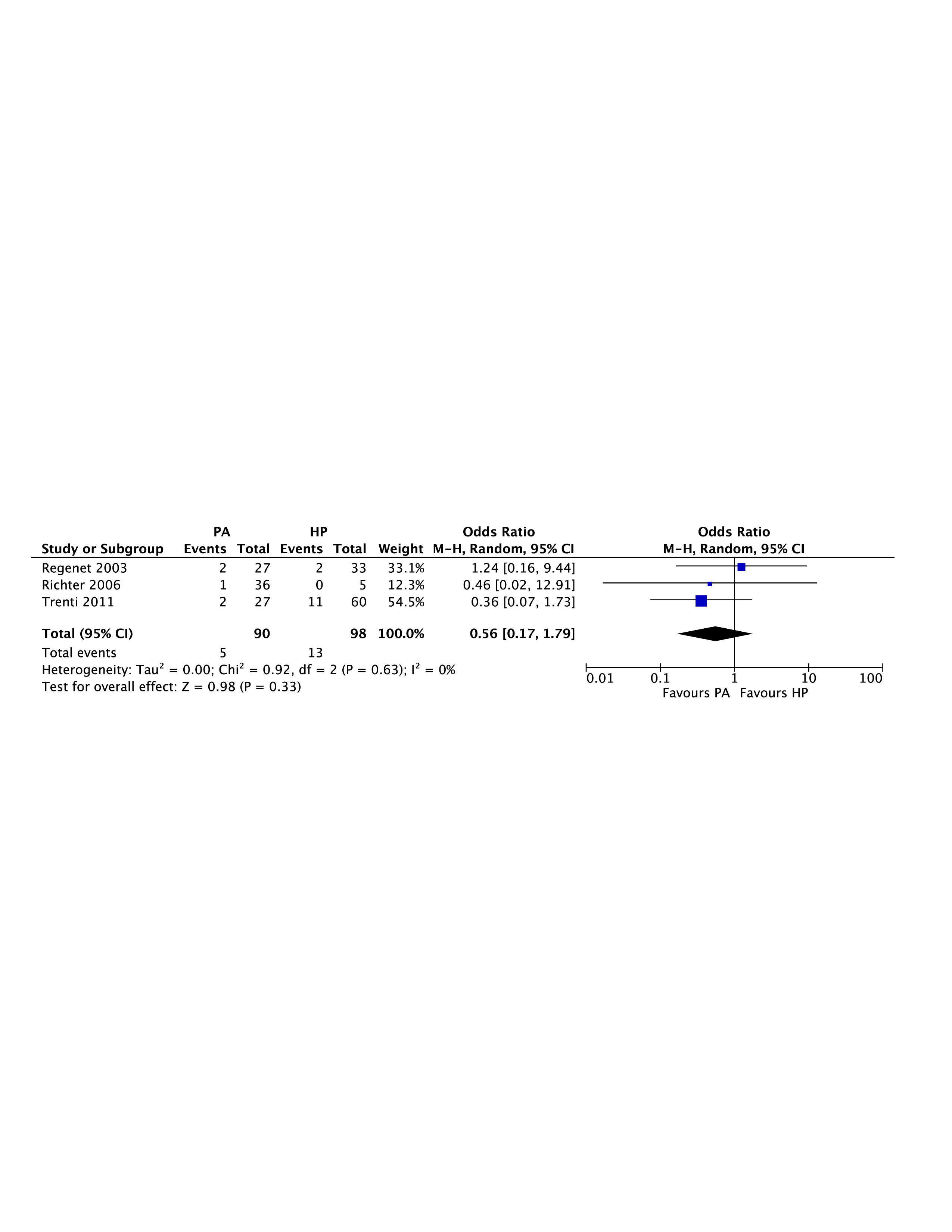

Supplement: Supplementary file 17 — Figure S3d (PNG 103 kb) [file 384_2020_3617_Fig12_ESM.png]

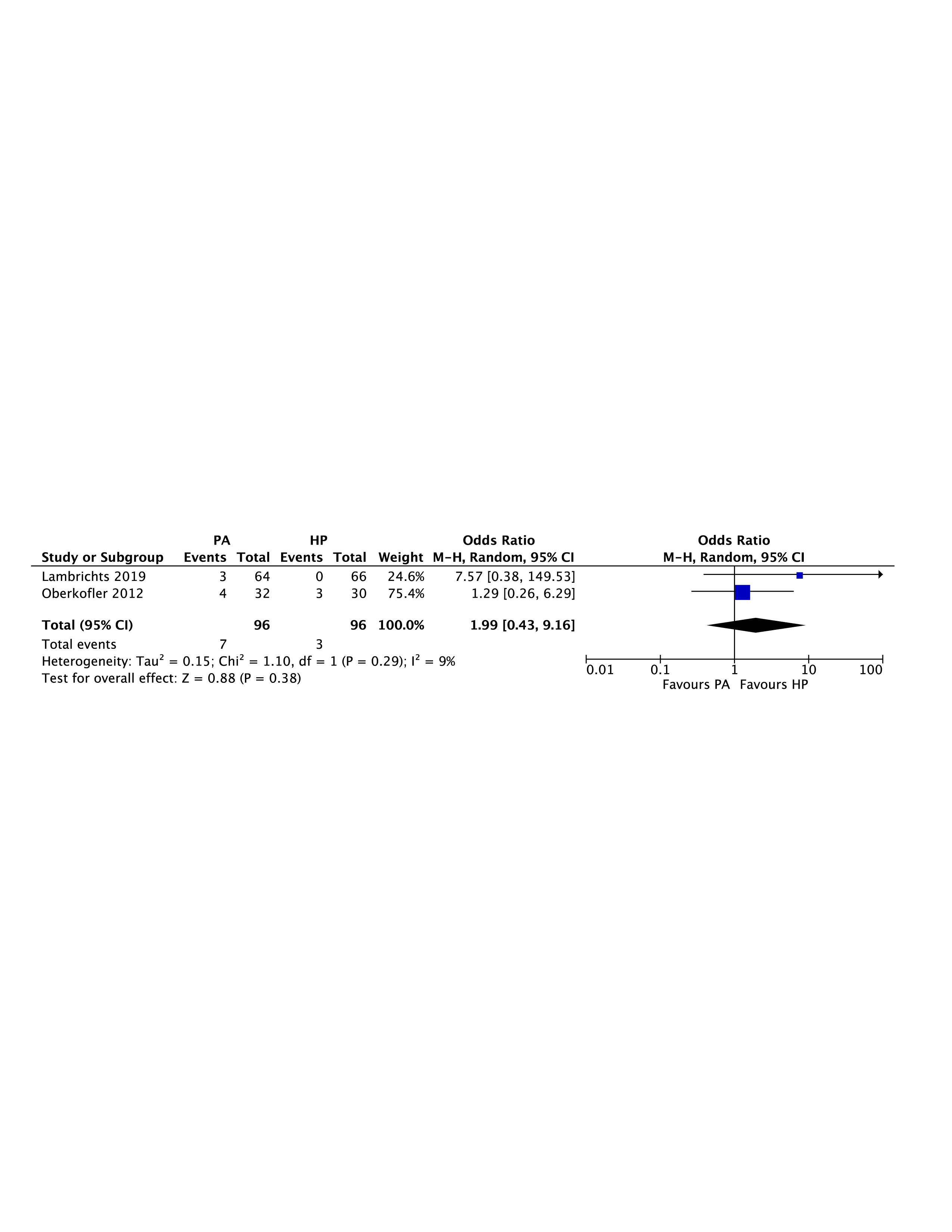

Supplement: Supplementary file 19 — Figure S3e (PNG 99 kb) [file 384_2020_3617_Fig13_ESM.png]

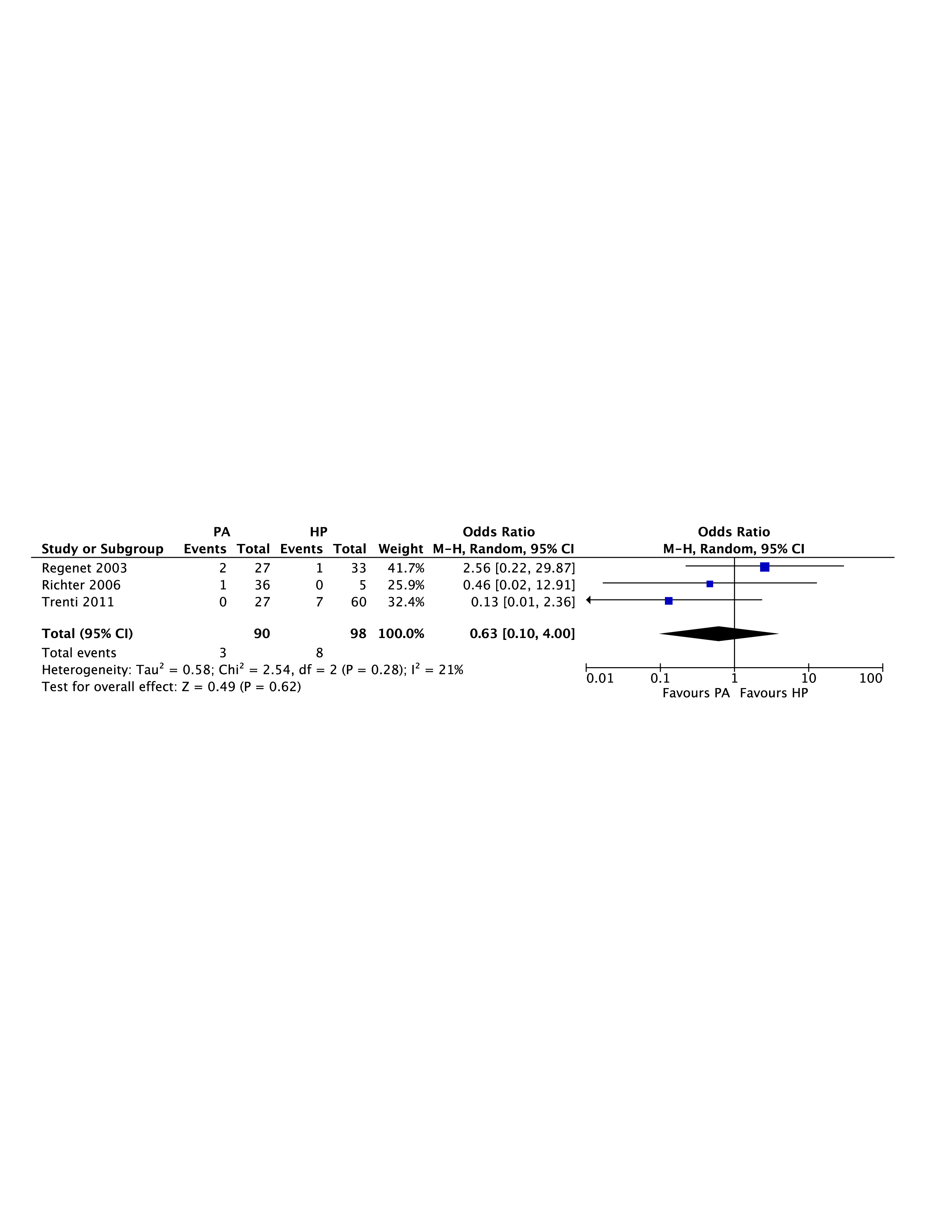

Supplement: Supplementary file 21 — Figure S3f (PNG 104 kb) [file 384_2020_3617_Fig14_ESM.png]

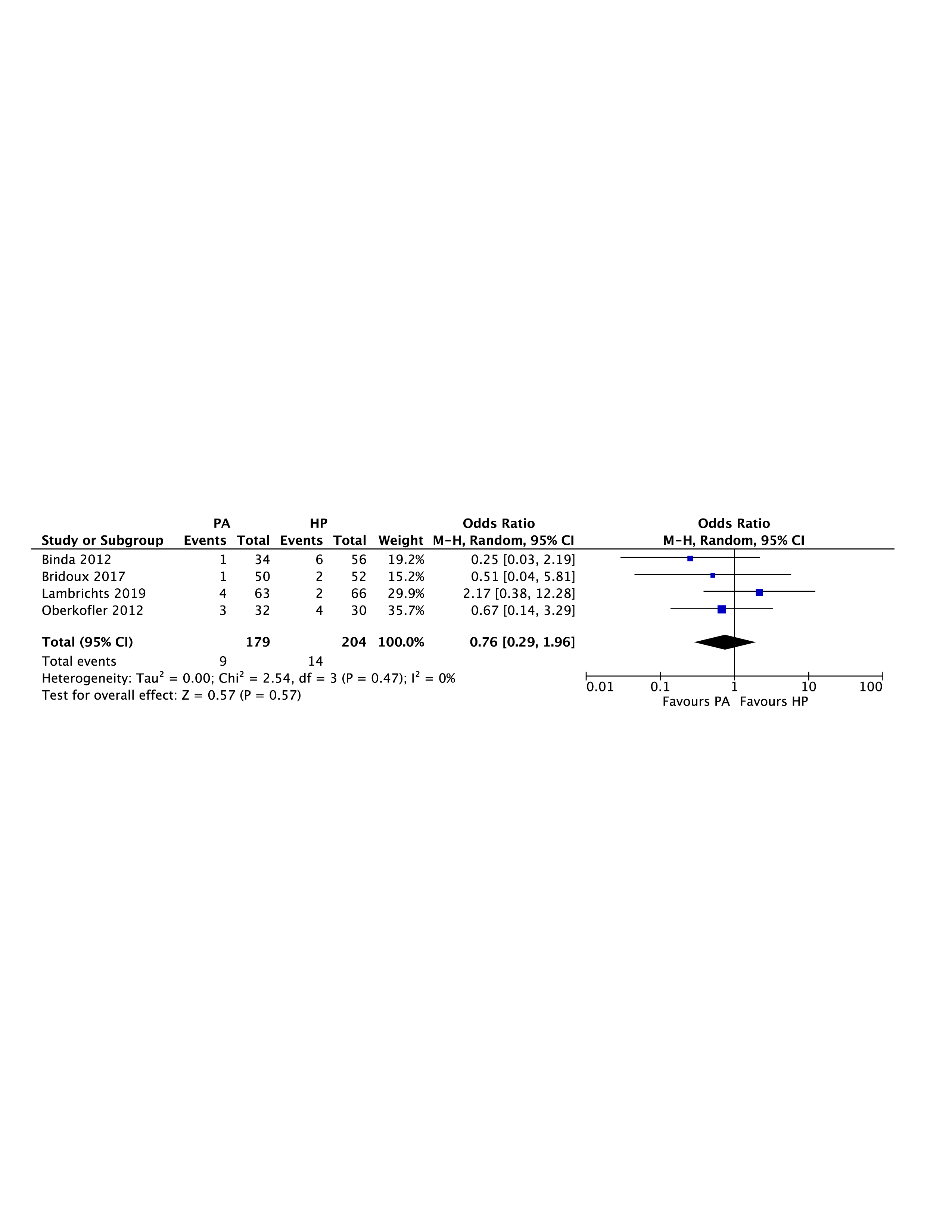

Supplement: Supplementary file 23 — Figure S4a (PNG 111 kb) [file 384_2020_3617_Fig15_ESM.png]

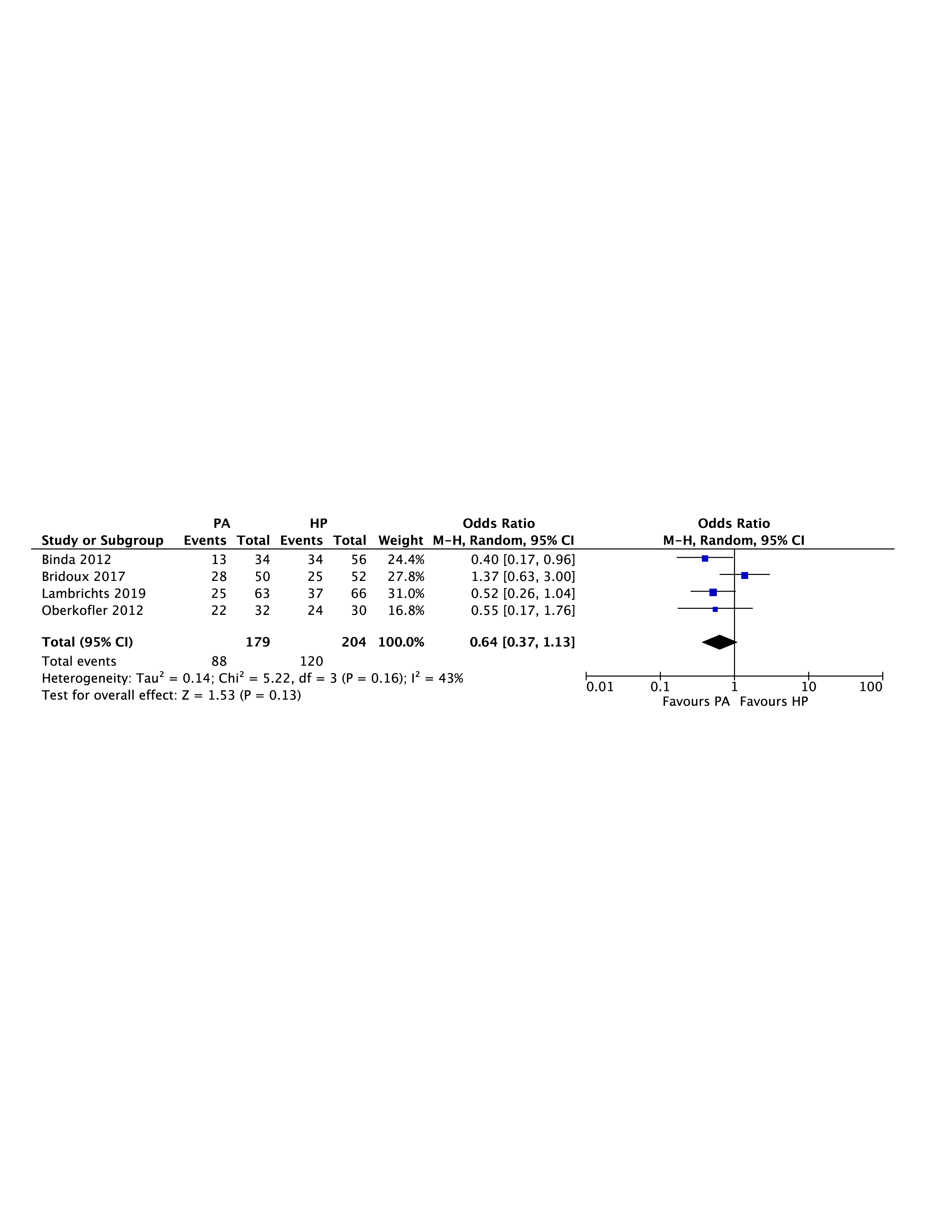

Supplement: Supplementary file 25 — Figure S4b (PNG 113 kb) [file 384_2020_3617_Fig16_ESM.png]

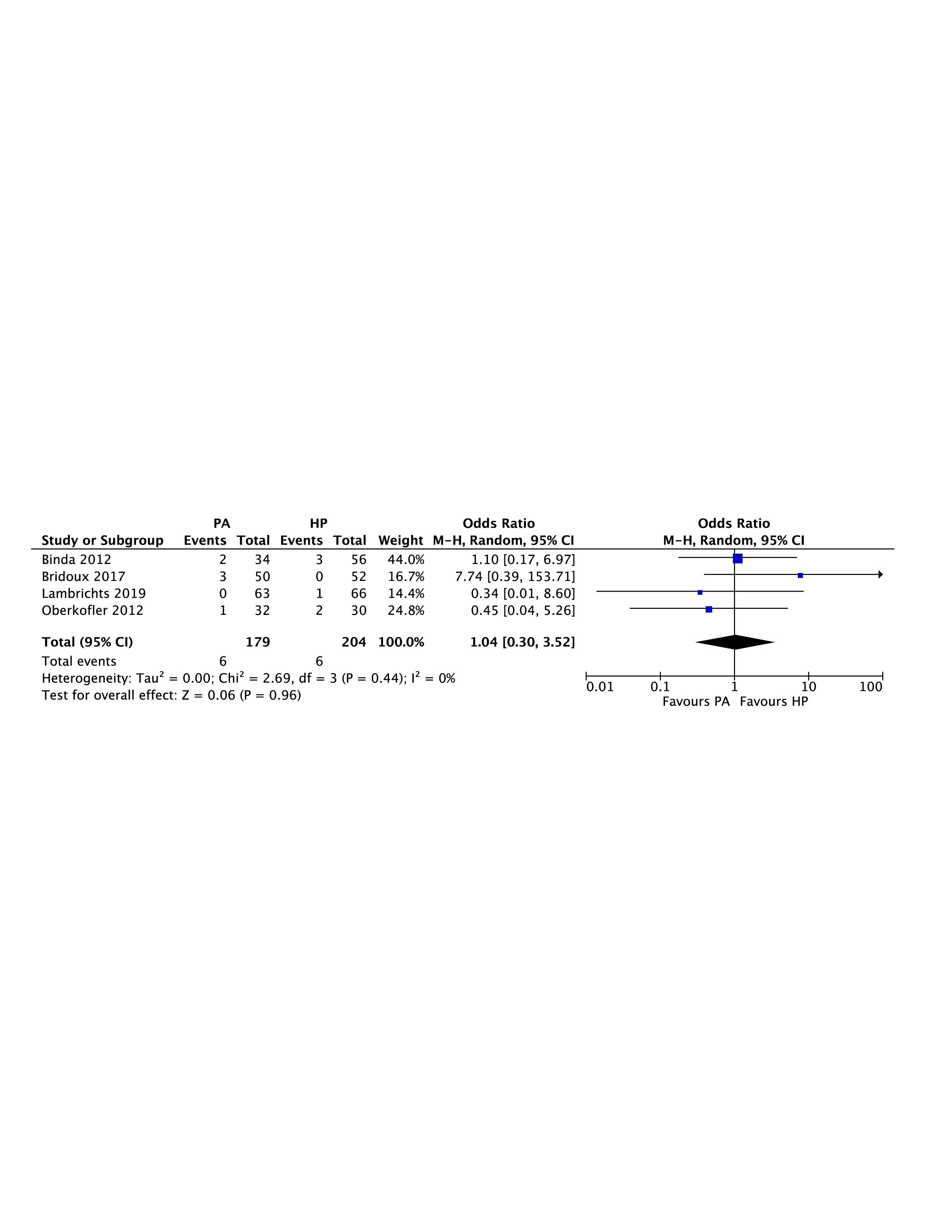

Supplement: Supplementary file 27 — Figure S4c (PNG 112 kb) [file 384_2020_3617_Fig17_ESM.png]
